# The German Auditory and Image (GAudI) vocabulary test: A new German receptive vocabulary test and its relationships to other tests measuring linguistic experience

**DOI:** 10.1371/journal.pone.0318115

**Published:** 2025-04-28

**Authors:** Sandra Bethke, Antje S. Meyer, Florian Hintz

**Affiliations:** 1 Max Planck Institute for Psycholinguistics, Nijmegen, Netherlands; 2 Radboud University, Nijmegen, Netherlands; 3 Philipps University, Marburg, Germany; 4 Center for Mind, Brain and Behavior, Philipps University Marburg & Justus Liebig University Giessen, Giessen, Germany; Hong Kong Baptist University, HONG KONG

## Abstract

Humans acquire word knowledge through producing and comprehending spoken and written language. Word learning continues into adulthood and knowledge accumulates across the lifespan. Therefore, receptive vocabulary size is often conceived of as a proxy for linguistic experience and plays a central role in assessing individuals’ language proficiency. There is currently no valid open access test available for assessing receptive vocabulary size in German-speaking adults. We addressed this gap and developed the *German Auditory and Image Vocabulary Test* (GAudI). In the GAudI, participants are presented with spoken test words and have to indicate their meanings by selecting the corresponding picture from a set of four alternatives. Here we describe the development of the test and provide evidence for its validity. Specifically, we report a study in which 168 German-speaking participants completed the GAudI and five other tests tapping into linguistic experience: one test measuring print exposure, two tests measuring productive vocabulary, one test assessing knowledge of book language grammar, and a test of receptive vocabulary that was normed in adolescents. The psychometric properties of the GAudI and its relationships to the other tests demonstrate that it is a suitable tool for measuring receptive vocabulary size. We offer an open-access digital test environment that can be used for research purposes, accessible via https://ems13.mpi.nl/bq4_customizable_de/researchers_welcome.php.

## Introduction

People acquire many words over the course of their lives. By the age of 20, the average native speaker of American English knows approximately 42,000 lemmas, and word knowledge accumulates further amounting to 48,000 lemmas on average by the age of 60. However, the variation between people is quite large and so the number of words known may range from 27,000 lemmas up to 52,000 lemmas [1,2]. Because people vary in which and how many words they know, vocabulary tests have been used in numerous contexts to capture the size and depth of individuals’ vocabulary. These applications range from educational assessments, where vocabulary size is used as an estimate for language development in children [3], to clinical contexts, where vocabulary knowledge is used as an estimate for cognitive functioning [4]. In general, an individual’s vocabulary size is an important proxy for their experience with a language. In language research, vocabulary is considered an estimate of general language proficiency [5]. There are numerous vocabulary tests available in different languages to assess and evaluate different aspects of an individual’s vocabulary knowledge. However, there is currently no standardized and openly available receptive vocabulary test for *German*-speaking adults. In the current study, we address this gap by introducing the *German Auditory and Image Vocabulary Test* (GAudI), a new test measuring receptive vocabulary in German-speaking adults, aged between 18 and 30 years. The GAudI is an adaptation of the *Dutch Auditory & Image Vocabulary Test* (DAIVT) by Bousard and Brysbaert [6], which is an open-access receptive vocabulary measure that has been validated in a sample of higher education students.

### Vocabulary size and linguistic experience

Psycholinguistic research has shown that large vocabularies benefit language comprehension and production. Specifically, larger vocabularies have been associated with better listening [7] and reading comprehension [8] and with faster word recognition. For example, individuals who knew many words were faster at carrying out a lexical decision task, where they had to indicate whether spoken or written stimuli were words of their language [9–11], than individuals with smaller vocabularies. Larger vocabularies also facilitate word production processes as indicated by shorter onset latencies in picture naming and verbal fluency tasks [12], and producing more words in spontaneous speech tasks [13]. A prominent theoretical account for these findings assumes that larger vocabularies are associated with increased precision and stability of lexical representations [11]. The acquisition of such large vocabularies depends on a variety of interacting variables, pertaining both to the individual learner’s skills and abilities and their language experience, i.e., the words they encounter. Children and adults differ in how quickly they acquire novel words [10,14], and this difference can be traced back to differences in domain-general skills such as working memory and processing speed. For instance, better phonological short-term memory has been found to lead to improved word learning and vocabulary size in children [15], while for adults, higher processing speed and non-verbal intelligence are beneficial for word learning [10]. These findings demonstrate how learning skills predict vocabulary learning.

Vocabulary size also depends on exposure; learners need to encounter a wide range of words in order to develop large vocabularies. This can be seen, for example, in the interplay of vocabulary size and word frequency. Individuals with a high degree of exposure to or experience with a language are less affected by word frequency because they have experienced more words, and also more infrequent words, than individuals with less exposure to the same language. Individuals with larger vocabularies therefore show smaller word frequency effects than individuals with smaller vocabularies [16], because the high degree of exposure to words stabilizes their lexical representations. Such stability of lexical representations is also known as the lexical entrenchment hypothesis [9]: lexical representations are more entrenched, robust [16], integrated [11], precise, or of higher lexical quality [17] in speakers with more language experience (i.e., greater vocabulary knowledge), which in turn enables faster lexical access.

Written texts contribute significantly to building such large vocabularies because they include more varied vocabularies than spoken language and a far higher proportion of relatively infrequent words [18–20]. Individuals with large vocabularies typically have had more exposure to written language. This is reflected in the association between vocabulary knowledge and measures of print exposure, such as the *Author Recognition Test* [21–23], which indicates that individuals who know many words typically know these words from frequent exposure to written materials. However, this written language experience also provides other benefits for individuals’ overall linguistic knowledge. Frequent readers are more familiar with syntactic constructions of varying grammatical complexity, such as complex noun phrases [24,25], passive as well as quantifier constructions [26], and syntactic structures including subordination [27]. This might be the case because such complex constructions are relatively frequent in written texts, and written language experience has been found to be a predictor of syntactic knowledge as measured in grammaticality judgement tasks [28]. In conclusion, vocabulary size has been found to be highly related to word comprehension and production processes and many aspects of linguistic experience, such as exposure to written text (print exposure) and knowledge of complex syntactic constructions.

### Assessing adults’ vocabularies

To assess an individual’s vocabulary size, several test formats and tasks have been used. Many of them were established in the context of language acquisition research and were therefore designed for children or young adults. The following review focuses on receptive vocabulary measures developed for adult language users.

One class of tests focusses on measuring knowledge of written word forms. In such tests, target words are presented in simple non-defining sentences followed by possible definitions of the word meaning. Participants are then asked to select the correct target definition out of these candidates. Examples include the *Vocabulary Size Test* [29] for English speakers and the *Receptive Multiple Choice Test* [7] for Dutch speakers. In the latter test, the four possible answers are accompanied by an option to choose “I really don’t know”. A German version of this class of tests exists, the *Receptive German 3 Vocabulary Size Test* [30]. However, it was normed in children between the age of 12 and 14. A similar test format is used in the *Wortschatztest (aktiv und passiv)* [31], the German version of the *Mill Hill Vocabulary Scale* [32], developed for teenagers and adults. Similar to the *Vocabulary Size Test* [29], participants are asked to choose the correct synonym of a written target out of six possible definitions.

A related format is used in tests such as the English *Vocabulary Levels Test* [33,34], which measures written word form knowledge of particular frequency levels of words (e.g., the 1,000 most frequent words, the second 1,000 words, etc.). Participants are presented with a list of six words and three definitions, and are instructed to map one word from that list to each of the three definitions (leaving three words unassigned). The definitions are either synonyms, paraphrases or gapped sentences of the matching word. Inspired by Laufer and Nation’s *Vocabulary Levels Test* [33], the Leipzig Institute for Test Research and Test Development (ITT) developed a *Receptive Vocabulary Size Test* [35], which is available in several languages including English, German, Dutch, French, Spanish, Italian and Chinese, among others. This test is designed in the same way as the original test, but measures how many of the most frequent 5,000 words of a language are known to the participant. Since it only features the most frequent words, this test is relatively easy and intended primarily for foreign language learners and children.

The tests discussed above measure written word recognition and the extent to which written word forms are connected to a known semantic concept. A related class of tests capitalizes more on word form recognition and less on semantics. Examples include Schmidt and Metzler’s *Wortschatztest* [36] and the *Mehrfachwahl-Wortschatz-Intelligenztest* [37,38], both available in German, in which participants have to identify the real word among four to six non-word distractors. Since real words have to be distinguished from non-words only, the meaning of the words does not necessarily have to be known in these tasks in order to identify the target.

Another frequently used test format for measuring receptive vocabulary size involves spoken target words that have to be mapped onto pictures representing the meaning of the target words. The most widely known implementation of this test format is the *Peabody Picture Vocabulary Test* (PPVT) [39] with more than 12,000 citations since publication. In this test, participants are instructed to select from a set of four alternatives the image that embodies the meaning of the spoken target. This test measures semantic knowledge and image-to-concept mapping, rather than form-meaning connections. No knowledge of written forms is needed to complete this task. The PPVT exists in multiple languages and was standardized in large samples: the original English version of the PPVT was normed in 3,540 English speakers between the ages of 2 and 90 years. The Dutch version of the PPVT (PPVT-NL) was normed twice, once in a sample of 1,746 children and teenagers between 2 and 15 years, and once in 1,164 adult Dutch speakers between 17 and 90 years of age [40]. There are also Spanish and French versions available, both of which are normed for children between 2 and 17 years. Similarly, the German version of the *Peabody Picture Vocabulary Test* (PPVT-DE) [37] was normed in 3,555 children and adolescents between the age of 3 and 16 years. That is, in contrast to its English and Dutch counterparts, the German version of the *Peabody Picture Vocabulary Test* has never been standardized in adult speakers. Additionally, the PPVT-DE is only available in an analogue pen-and-paper format distributed by Pearson Clinical, which renders the PPVT-DE impractical for administration in larger groups.

Recently, Bousard and Brysbaert [6] developed a Dutch version of a picture-based vocabulary test using the same paradigm as the PPVT, the *Dutch Auditory & Image Vocabulary Test* (DAIVT). This test was validated in a sample of higher education students and is freely accessible online. However, there currently exists no standardized and openly available receptive vocabulary test for German adults. Picture-based vocabulary tests do not require reading and written word form knowledge, but focus on the mapping of semantic concept to image. Thus, we followed Bousard and Brysbaert’s [6] example and developed a German vocabulary test using a picture-based test format. In the present paper, we report the development and validation of the *German Auditory and Image Vocabulary Test* (GAudI), a receptive vocabulary test specifically designed for German speakers.

### Developing the GAudI

Developing reliable test instruments is generally a time- and labor-intensive process, in which item sets have to be carefully curated, tested and revised multiple times. This process becomes particularly challenging when developing tests that are intended to be comparable across languages, as the test items need to be carefully matched for any characteristics that might affect their difficulty. In tests of vocabulary size one would, for instance, aim to match the items in terms of frequency, word class, concreteness and animateness. Margaretto and Brysbaert [42] recently highlighted that an efficient way of developing parallel tests for different languages is to start by translating test items from one language to another. This works particularly well when the languages of interest are linguistically similar and spoken in similar cultural contexts. Margaretto and Brysbaert used this cross-linguistic translation approach to develop language skills tests for high-achieving Spanish-speaking adults (i.e., mainly university students), based on existing tests for speakers of English. These language skills tests included a vocabulary test, for which they found that almost all items could be directly translated. For only four items a direct translation was not possible, because a translation equivalent to an English word did not exist in Spanish or because the Spanish translation differed greatly in frequency from the English word.

Mimicking Margaretto and Brysbaert’s approach, we used the *Dutch Auditory & Image Vocabulary Test* (DAIVT) [6] as a template and started out by translating the Dutch items to German. As described in detail below, we then modified the resulting set of words where necessary. Especially for highly educated adult samples, for whom the GAudI was intended, curating reliable item sets is challenging, as items have to be rare in order for the test to elicit considerable response variability between test takers. Our aim in selecting the words was to maintain all of the pictorial materials and to find German test words that matched the Dutch ones as well as possible in frequency. This should lead to a German test that would be roughly equivalent to the Dutch test in terms of the overall difficulty of the items and in the spread of item difficulty.

**Fig 1:**
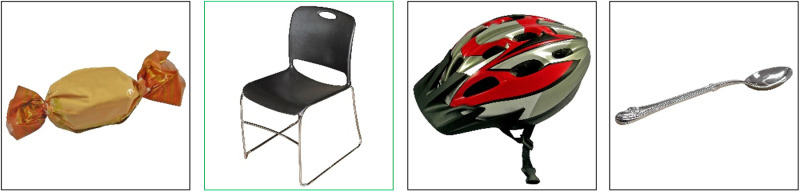
Example trial of the GAudI. The cue is “Stuhl” (chair). The green coloring marks the correct target image. Images reprinted from [6] under a CC BY license, with permission from Marc Brysbaert, original copyright 2021.

The GAudI builds on the previous work by Bousard and Brysbaert [6] and is a picture-based receptive vocabulary test. On each trial, participants are presented with a spoken word and four images – one target and three distractors – and are instructed to select the image that matches the target (Fig 1). As described above, the selection of items was based on the DAIVT, for which items were derived based on the English and Dutch *Peabody Picture Vocabulary* *Tests* [39,40] as starting points. For each of the items, frequency values (SUBTLEX-NL [43]) and word prevalence [44] were obtained. Based on these values, items were selected that (1) were neither extremely difficult (low frequency) nor very easy (high frequency) words; were of (2) different word types (nouns, adjectives, and verbs); (3) represented a range of different concepts (such as objects, actions, sceneries, and more abstract words), and were of (4) a range of different semantic categories. The mean Zipf frequency of the targets was 2.06 (range = 1.36-3.80) [6]. Images for these items were mainly taken from the Bank of Standardized Stimuli (BOSS) [45] and another picture database by Moreno-Martínez and Montoro [46], while some images were taken from open access picture websites or created from scratch.

To develop the GAudI, all 90 items of the DAIVT were translated from Dutch to German using PONS [47]. Translations were verified using Google Translate [48]. Similar to the item selection described for the DAIVT, frequency values for the translated items were obtained from SUBTLEX-DE [49]. Native speakers of German judged the frequency and usability of the items, i.e., they decided whether the frequency values resembled their usage of the word and whether the meaning was retained after translation. If the translation was judged as fitting, the item was retained in the item set. If there was no direct translation, (1) a word was chosen that translated the item more loosely, (2) the word class of the original item was changed (e.g., from noun to verb), (3) the focus of the target image was shifted to a different aspect of the picture, or (4) a distractor image was used as the target and the original target image served as a distractor. Six new items were added, which were taken from a previous version of the DAIVT (for more detail, see the description of the test development in [6]), resulting in an initial set of 96 items. No changes were made to the image materials.

The initial version of the GAudI was tested on 84 participants as part of a larger study at the Max Planck Institute for Psycholinguistics [50]. Nineteen out of the 96 items showed no response variability or poor discrimination parameters and were revised. For example, instead of “empört” (*outraged*), “indigniert” (*indignantly*) was used for the same experimental image. Another item, “ausbaggern” (*to excavate*) was omitted, since the four images appeared to depict very similar actions and made it difficult for participants to identify the target image. This resulted in a revised set of 95 items, which was tested on a different sample of 91 participants. Revisions were made to the second version of the test to finalize the item set: five out of the 95 items showed bad discrimination parameters and were omitted from the final item set. Another six items showed no response variability. Five out of these six items were omitted, while one of these items was kept: “Spachtel” (*scraper*) was retained in the item set as it had elicited some response variability in the first version of the GAudI. These revisions reduced the item set to 85 items.

The final version of the GAudI contained 85 items, of which 53 were nouns, 19 were adjectives and 13 were verbs. The items represented concrete (e.g., “Hellebarde”, *halberd*) as well as abstract (e.g., “prekär”, *precarious*) concepts. All items and distractors were depicted as coloured photographs, taken from the final as well as previous versions of the DAIVT (kindly provided by Marc Brysbaert, refer to [6] for more detail). On average, the items were 8.51 characters long (range = 4-15) and contained 3.06 syllables (range = 1-6). Frequency values were retrieved from SUBTLEX-DE [49]. Mean Zipf frequency was 2.25 (range = 1.66-3.30). These frequency values match the item set of the DAIVT, with a mean Zipf frequency of 2.06 (range = 1.36-3.80) [6]. Note that Zipf frequency values below 3 are generally considered low frequency items. For 31 items, the frequency values could not be determined as they were not listed in SUBTLEX-DE. Thus, additionally the *Häufigkeitsklassen* (frequency classes) provided by the *Projekt Deutscher Wortschatz* (Project German Vocabulary) of Leipzig University [51] were retrieved for all 85 items. A high frequency class value means that a word is low in frequency, while low numbers indicate high frequency (e.g., a word in frequency class 9 is about twice as frequent as a word in class 10; values above 20 refer to very infrequent words [51]). The mean frequency class value was 17.94 (range = 11-24). Seven items did not have an entry. To cover these items as well, the frequency designations of the *Digitales Wörterbuch der deutschen Sprache* (DWDS) [52] were retrieved, which had entries for all items of the test. Similar to the frequency classes of the *Projekt Deutscher Wortschatz*, the DWDS uses frequency levels from 0 to 6, with 0 indicating low frequency and 6 indicating high frequency. The mean frequency level value across all words was 1.49 (range = 1-3).

All items were spoken by a male native speaker of German and recorded in a soundproof booth. The recordings were on average 1022.89 ms long (range = 715-1393 ms).

### Administration and validation of the GAudI

The present paper had two goals: First, we report the development and administration of the *German Auditory and Image Vocabulary Test* (GAudI), a receptive vocabulary test specifically designed for German adult speakers. The GAudI was tested on 168 German-speaking participants between the ages of 18 and 30 years, since this age range reflects the samples in many psycholinguistic experiments. The sample size was similar to the one reported for its Dutch counterpart, the DAIVT, which was tested on two samples: 49 Belgian first year university students aged between 17 and 22 years, and 108 Dutch speakers aged between 17 and 26 years. The GAudI consists of 85 experimental items. Participants’ accuracy, measures of central tendency, skewness and kurtosis of the distribution, and internal consistency are provided.

The second goal was to report evidence for the validity of this newly developed vocabulary test by comparing it to the results reported for the DAIVT, as well as by reporting the relationship of the GAudI with other measures of linguistic experience. Since the GAudI was developed based on the DAIVT, both tests were expected to elicit comparable internal consistency scores and correlations to other tests measuring linguistic experience. Bousard and Brysbaert [6] related the DAIVT to two other receptive vocabulary tests, the PPVT-NL and the multiple choice test developed by Andringa et al. [7]. Since there are no comparable vocabulary tests for German adults, we compared the GAudI to the PPVT-DE as a measure of receptive vocabulary and to the German *Author Recognition Test* (GART) [53] as a measure of print exposure. In order to provide an additional layer of validity for the GAudI, we also compared it to other aspects of linguistic experience: two tests of semantic knowledge (antonyms) using production tasks and one test of grammatical knowledge. The sample of 168 participants therefore completed five tests in addition to the GAudI.

In the PPVT-DE, participants were asked to select the image matching the auditory target out of a set of four candidate images. The task was thus the same as for the GAudI. In line with the validation of the DAIVT, we included the PPVT-DE in the present study as a reference measure of receptive vocabulary, knowing that its items would probably be too easy for our participants. The GART measures knowledge about authors (print exposure) by asking participants to identify names of existing fiction writers (e.g., Agatha Christie).

To measure access to word meanings in a production task as an aspect of productive vocabulary, participants completed two newly developed antonym production tests. In these tests, participants were visually and auditorily presented with cue words and were instructed to produce their antonyms. In the untimed antonym production test, participants had as much time as they wished to produce the antonym. This version measures the accuracy with which participants produce an antonym. In the timed antonym production test, participants were asked to respond as quickly as possible, and there was a time limit of four seconds to respond. This version measures how long it takes participants to access and produce an antonym. Both antonym production tests were based on an existing Dutch antonym production test [10,54]. That test consists of 25 cues for which participants were instructed to produce their antonym. The mean Zipf frequency for the Dutch targets was 3.74 (range = 1.70-5.00). The prevalence values for the Dutch target words ranged from 0.85 to 1.00 (M = 0.98, SD = 0.04). The internal consistency measure of Cronbach’s alpha was 0.48 [54]. For the antonym production tests used in the current study, all of the 25 Dutch cue and target pairs were translated into German. Antonym pairs in which the target contained (parts of) the cue were omitted (e.g., *legal* – *illegal*). Additionally, translations with more than one obvious antonym were omitted. Ten items were retained, and 30 new antonym pairs were added after being judged by two German native speakers for their suitability. For all of these antonym pairs, Zipf frequencies were derived using SUBTLEX-DE [49]. The 40 items were then divided into two sets of 20 cue-target pairs featuring a range of high and low frequencies. One of these sets was used for the untimed antonym production task, while the other was used for the timed antonym production task.

Furthermore, participants completed a newly developed book language grammar test to assess their grammatical knowledge. Here, participants were asked to perform grammaticality judgements. This grammar test was modelled on an existing test by Hubers et al. [55], designed to test violations of grammatical norms that are often only judged as ungrammatical by language teachers and language purists (“school book language”), but are not commonly considered ungrammatical. Hintz et al. [54] adapted this test and administered grammatical categories that Dutch speakers often find difficult to use correctly (e.g., personal pronouns or comparatives).

For the German book language grammar test, we applied the same principles and selected six grammatical categories that German native speakers find difficult to use: (1) prepositions requiring genitives such as “Ein Weg in die Stadt verläuft links des Hauses” (*A path to the city runs to the left of the house*); (2) verbs requiring genitives such as “Das Stipendium entledigte sie all ihrer finanziellen Sorgen” (*The stipend rid her of all her financial worries*); (3) word order in subordination vs. coordination, as in “Karl hatte große Pläne fürs Wochenende, weil er nicht auf die Kinder aufpassen musste” (*Karl had big plans for the weekend, because he did not have to watch the children*); (4) subject-verb number agreement, as in “Die Lehrerin der Kinder spricht Englisch und Deutsch” (*The teacher of the children speaks English and German*); (5) comparatives like “Je mehr Philip arbeitet, umso höher ist sein Verdienst” (*The more Philip works, the higher his salary*); and (6) indirect speech as in “Tanja merkte an, ihr Freund komme später von der Arbeit zurück” (*Tanja remarked her boyfriend would be back from work later*). While violations of these grammatical rules may commonly be considered acceptable (e.g., using certain prepositions or verbs with the dative instead of the genitive), these rules are typically observed in (fiction and non-fiction) books and are described in standard German reference grammars (e.g., [56]). Thus, good knowledge of these rules may be seen as indicative of a high degree of familiarity with book language, reflecting another layer of linguistic experience.

As discussed above, we expected receptive vocabulary to be related to other aspects of linguistic experience. We therefore predicted performance on the GAudI to correlate with performance on all other tests tapping into linguistic experience. One might expect to see the strongest correlation between the GAudI and the other receptive vocabulary test, the PPVT-DE, since both tests measure receptive vocabulary size and have the same test format. However, the PPVT-DE has been normed for children and young adults [41] and should thus be fairly easy for our adult sample, which may reduce the correlation with the GAudI. We expected both receptive vocabulary tests to correlate strongly with the untimed antonym production test as a measure of productive vocabulary, since larger vocabulary size relates to more comprehensive semantic knowledge (i.e., more robust lexical representations) [57]. Additionally, we predicted the GAudI and PPVT-DE to correlate strongly with the GART: the more individuals are exposed to written language, the more words they should know. Relatedly, we expected the GAudI to correlate positively with the book language grammar test. We expected the weakest correlations between the timed antonym production and all other tests, because it was the only one including a time constraint. Since the GAudI was developed based on the DAIVT, internal consistency measures as well as correlations to the PPVT-DE were expected to be comparable to the results reported for the DAIVT.

## Method

### Participants

Simple correlations have been shown to stabilize at a sample size of 161 participants [58]. Guided by this number and paralleling the sample size of the DAIVT (n = 157) [6], we collected data from 168 German-speaking adults (132 female, 36 male) aged between 18 and 30 years (mean age = 23.71). Data collection took place online between May 2023 and January 2024. All participants were recruited online, mainly via Social Media platforms and mailing lists. They provided written informed consent and were paid 10,00 € for participation. One-hundred thirty-four participants were students in higher education. The study received ethical approval from the Social Sciences Ethics Committee of Radboud University (ECSW-LT-2022-1-20-47358) on 22.01.2022.

### Procedure

The six tests were administered online using Frinex, a programming environment developed by the technical group at the Max Planck Institute for Psycholinguistics [59]. The order of tests was: GAudI, GART, antonym production (untimed), antonym production (timed), book language grammar, PPVT-DE. The order of tests as well as the order of trials within each test were the same for each participant to eliminate potential order effects on participants’ performances [60].

The study was preceded by a participant questionnaire and a microphone test. The completion of all tests took approximately 40-50 minutes.

#### German Auditory and Image Vocabulary Test (GAudI).

Participants were presented with a spoken word and four images: one target image and three distractor images. Participants were instructed to select the target image that best fitted the spoken word by mouse-click (Fig 1). Participants could play the spoken word as often as they wanted by pressing the spacebar. There was no time limit and the next trial was initiated by selecting one of the images. The test took on average 15 minutes to complete. The test consisted of one practice and 85 test trials (S2 Table). Word frequencies were retrieved using SUBTLEX-DE [49], the *Projekt Deutscher Wortschatz* [51] and the *Digitales Wörterbuch der deutschen Sprache* (DWDS) [52]. Mean Zipf frequency was 2.25 (SD = 0.51, range = 1.66-3.30). The mean frequency class value was 17.94 (range = 11-24). The mean frequency level value derived by the DWDS was 1.49 (range = 1-3). As in the DAIVT, trials were pseudo-randomized in advance, i.e., items were not displayed in order of increasing difficulty. Participants’ accuracy was the performance indicator.

#### German Author Recognition Test (GART).

This test was developed and validated by Grolig et al. [53] and originally split into two parallel test forms. The authors report that the mean scores were almost identical for both forms (M = 0.46, SD = 0.24 for the first form; M = 0.49, SD = 0.25 for the second form). The high split-half reliability for both forms (r = 0.94; r = 0.95) indicated that both are equally reliable [53], such that for the current study, both test forms were combined into one. Participants, thus, identified authors in a list of 125 names, of which 75 were existing fiction writers (e.g., Agatha Christie) and 50 were foils. Existing authors and foils were listed in pseudo-random order and the number of existing authors was not known to the participants. The performance indicator was the proportion of correctly identified authors minus the proportion of foils incorrectly selected.

#### Antonym production test (untimed).

After the presentation of a fixation cross for 500 ms, participants heard and simultaneously saw a cue word. The written word remained on screen until the trial ended. Participants were instructed to produce the cue’s antonym (e.g., “hot” when having heard and read “cold”). Their spoken response was recorded and participants could proceed to the next trial via button-click. The test consisted of three practice and 20 test trials (S3 Table). The 20 cue words consisted of nine nouns, nine adjectives and two verbs of varying frequency. Word frequencies were retrieved using SUBTLEX-DE [49], the *Projekt Deutscher Wortschatz* [51] and the *Digitales Wörterbuch der deutschen Sprache* (DWDS) [52]. Mean Zipf frequency was 3.55 (range = 1.66-5.79). The mean frequency class value of the *Projekt Deutscher Wortschatz* was 12.65 (range = 5-20). The mean frequency level value of the DWDS was 3.1 (range = 2-5). Trials were pseudo-randomized in advance. Spoken responses were coded offline using Praat [61]. Participants’ accuracy was the performance indicator.

#### Antonym production test (timed).

The procedure was the same as for the untimed antonym production test, but with a time limit of four seconds to respond. The next trial started immediately after these four seconds. The test consisted of three practice and 20 test trials (S4 Table). The 20 cue words consisted of seven nouns, 10 adjectives, two verbs and one adverb. Word frequencies were retrieved using SUBTLEX-DE [49], the *Projekt Deutscher Wortschatz* [51] and the *Digitales Wörterbuch der deutschen Sprache* (DWDS) [52]. Mean Zipf frequency for the cue words was 4.01 (range = 2.13-5.23). The mean frequency class value derived by the *Projekt Deutscher Wortschatz* was 11.1 (range = 7-19). The mean frequency level value of the DWDS was 3.55 (range = 2-5). The overall frequency values were slightly higher than for the items in the untimed antonym production test. This was done to ensure that participants knew the cues and the targets and could give a response within the time limit. Trials were pseudo-randomized in advance. Spoken responses were coded offline using Praat [61]. The performance indicator was participants’ average onset latency for correctly named trials. Reaction times were trimmed from 300 ms to 3000 ms (i.e., latencies beyond these limits were excluded) and log-transformed. In order to align the dependent variable of this test to the other performance indicators, the reaction times were inverse-coded (multiplied by -1), so that higher scores represent better performance. Participants who retained minimally 80% of the data after excluding incorrect and very fast/slow responses were included in the analysis.

#### Book language grammar test.

Participants listened to spoken sentences featuring six morpho-syntactic categories with ambiguous grammaticality status known to be difficult for adult speakers of German: (1) prepositions requiring genitives, (2) verbs requiring genitives, (3) word order in subordination vs. coordination, (4) subject-verb number agreement, (5) comparatives and (6) indirect speech. Each of these categories consisted of eight items. Four out of these eight were considered grammatical German sentences in standard German reference grammars (e.g., [56]), while the other four were considered ungrammatical sentences. Participants indicated for each of the sentences whether they thought it was a grammatical German sentence by clicking on a box on screen (“Richtig” on the right-hand side for grammatical, “Falsch” on the left-hand side for ungrammatical). Responses could be given during or after the presentation of the sentence. Clicking on one of the boxes started the next trial. The test consisted of two practice and 48 test trials (S5 Table). Trials were pseudo-randomized in advance. The performance indicator was the proportion of correct responses.

#### Peabody Picture Vocabulary Test (PPVT-DE).

Participants heard a spoken word and selected the picture corresponding to its meaning among four alternatives. Participants could listen to the word as often as they wanted. By selecting one of the four images via mouse-click, they started the next trial. The original version of the PPVT-DE comprises 228 items categorized in 19 sets of increasing difficulty [41]. For the current study, the first 13 sets were omitted for being too easy for adult speakers. The version used in this study was shortened to the 72 trials belonging to the last six sets. Each set comprised 12 trials of roughly the same difficulty, while each set was more difficult than the previous one. The test ended when a participant made more than eight errors within one set or when the last test item was responded to. As in [6], we chose to operationalize the performance indicator as the difference between the item number of the last item responded to and the number of errors made.

## Results

Table 1 displays the descriptive statistics for the six tests. Four of the 168 participants did not complete all tests: Two participants had missing data for the GART and both antonym production tests, three participants had missing data for the book language grammar test and four participants had missing data for the PPVT-DE.

**Table 1:** Descriptive statistics for the six tests in the order of appearance.

Test	DV	n	mean	sd	skew	kurt	Min	max	IC
GAudI	accuracy	168	0.75	0.12	-0.29	-0.74	0.46	0.95	0.88
German Author Recognition Test	score	166	0.28	0.18	0.75	-0.03	0.00	0.87	0.94
Antonym Production Test (untimed)	accuracy	166	0.86	0.11	-1.14	4.91	0.35	1.0	0.62
Antonym Production Test (timed)	log RTs	166	-3.1	0.06	0.13	-0.31	-3.25	-2.92	0.92
Book Language Grammar Test	accuracy	165	0.72	0.09	-0.48	0.49	0.38	0.94	0.68
PPVT-DE	score	164	215	6.17	-1.2	2.25	187	227	0.8

DV=dependent variable, n=number of participants, sd=standard deviation, skew=skewness, kurt=kurtosis, min=minimum, max=maximum, IC=internal consistency.

**Fig 2:**
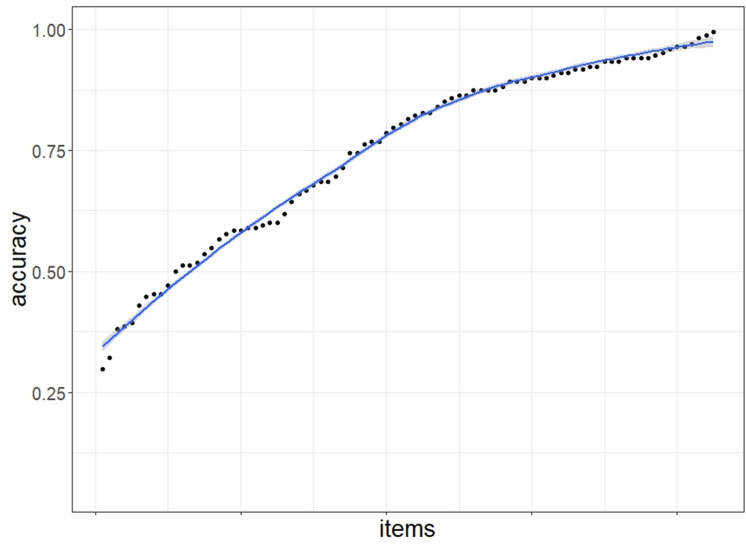
Distribution of items by mean accuracy for GAudI.

The mean accuracy for the GAudI was 0.75 (SD = 0.12, range = 0.74-0.95) and the distribution of the data suggests high response variability among participants (skewness = -0.29, kurtosis = -0.74). This indicates that the test was neither too easy nor too difficult for the current sample. The mean accuracy per item was 0.75 (SD = 0.19, range = 0.3-0.99) with a skewness of -0.61 and kurtosis of -0.88. The reliability measure of Cronbach’s alpha suggests that the GAudI had excellent internal consistency (α=0.88). Fig 2 plots the distribution of items by accuracy. The almost linear increase of the accuracy data suggests that the response range (from 0.3 to 0.99) was well balanced and that the item difficulty increased gradually.

The range of mean accuracies, mean scores and mean reaction times for the other tests in the current study also suggest variability among the participants (Table 1), which was reflected in the normal distribution of the participant scores. The distributions of the untimed antonym production test and the PPVT-DE were left-skewed, indicating that the majority of participants scored high on both tests. Internal consistency values ranged between 0.62 and 0.94 with the lowest values for the untimed antonym production and book language grammar tests (0.62 and 0.68), respectively.

**Fig 3:**
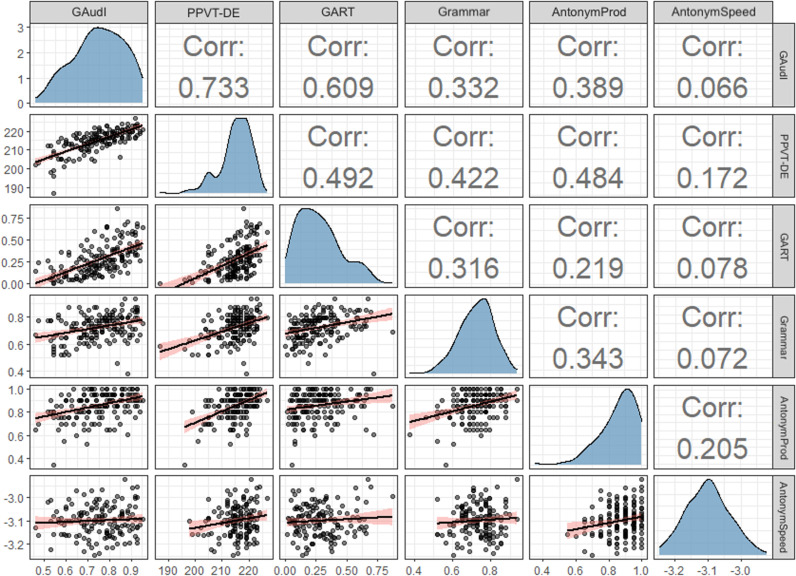
Correlation matrix with distributions and densities of the six tests. Grammar=book language grammar test, AntonymProd=antonym production test (untimed), AntonymSpeed=antonym production test (timed). Corr=Pearson correlation coefficient.

To assess the relationships between the GAudI and the other five tests tapping linguistic experience, we performed Pearson’s correlation analyses. We applied multiple-comparison correction using Bonferroni adjustments [62] (i.e., dividing the α-level of 0.05 by the number of comparisons, n = 15). The adjusted p-value was 0.003. As expected, the GAudI correlated positively with all tests (Fig 3). The correlation with the timed version of the antonym production test failed to reach significance level (r = 0.07, p = 0.417).

The strongest correlations were seen between GAudI and PPVT-DE (i.e., both receptive vocabulary tests, r = 0.73, p < .001) and between the GAudI and the German *Author Recognition Test* (r = 0.61, p < .001). The correlations between the GAudI and the untimed antonym production test (r = 0.39, p < .001) and between the GAudI and the book language grammar test (r = 0.33, p < .001) had moderate strength.

Furthermore, we observed that the PPVT-DE correlated positively with the German *Author Recognition Test* (r = 0.49, p < .001), with the untimed antonym production test (r = 0.48, p < .001) and with the book language grammar test (r = 0.42, p < .001). The German *Author Recognition Test* and the grammar test were also significantly correlated (r = 0.32, p < .001) (Fig 2). The untimed antonym production test correlated positively with both receptive vocabulary and the book language grammar tests (r = 0.34, p < .001). Although the correlation between the German *Author Recognition Test* and the untimed antonym production test was close to statistical significance (r = 0.22, p = .005), the r of 0.22 indicates weak-to-moderate strength.

Additionally, we calculated the correlations corrected for reliability. This procedure is often called correction for attenuation and serves as an indicator for what the correlation between two measures would be if both had perfect reliability [63]. The correlation corrected for reliability was 0.87 between GAudI and PPVT-DE, 0.67 between GAudI and GART, 0.43 between GAudI and the book language grammar test, 0.53 between GAudI and the untimed antonym production test, and 0.07 between GAudI and the timed antonym production test.

We performed an exploratory factor analysis (EFA) using JASP [64] to determine whether all six tests measure the same underlying construct assumed to reflect linguistic experience. The Kaiser-Meyer-Olkin (KMO) test [65] for sampling adequacy showed that all variables had a KMO measure above 0.6. Based on the KMO and Bartlett’s test [66] for sphericity (χ2(15) = 299.64, p < .001), the data was deemed suitable for a factor analysis. We used maximum likelihood as a factoring method. The number of factors was set to be based on parallel analysis (based on PC), and the rotation was set to oblique (promax). Table 2 displays the EFA loadings for all six tests. With the exception of the timed antonym production test, all tests loaded on one factor with loadings ranging between 0.46 and 0.86 (cf. [67]). Both receptive vocabulary tests, the PPVT-DE and GAudI, had the highest factor loadings (0.86 and 0.85), followed by the *Author Recognition Test* (0.63), untimed antonym production test (0.51) and book language grammar test (0.46) (Table 2). The timed antonym production test did not load strongly on the factor (0.15). These findings are in line with the correlations reported above.

**Table 2:** EFA component loadings for all six tests.

Test	Factor 1	Uniqueness
PPVT-DE	**0.86**	0.26
GAudI	**0.85**	0.27
German Author Recognition Test	**0.63**	0.6
Antonym Production Test (untimed)	**0.51**	0.74
Book Language Grammar Test	**0.46**	0.79
Antonym Production Test (timed)	0.15	0.98

Factor scores above 0.4 are in bold print.

To provide another layer of validity, we performed a Rasch analysis of the GAudI results to assess item fit (i.e., determine whether the individual items contribute to measuring the latent trait) using the mirt package [68] in R. The obtained RMSEA value of 0 suggests that the data fit the model well. Infit mean square values for the item set ranged from 0.82 to 1.22 (M = 0.99). Outfit mean square values ranged from 0.48 to 1.39 (M = 0.93). All infit and outfit mean square values were within the 0.5 and 1.5 range [69] and, thus, considered productive for measurement (S6 Table). The results are available in the Supporting Information (S6 Table and S7 Fig).

## Discussion

While there are numerous tests in different languages available to assess individuals’ vocabulary size, there currently exists no standardized receptive vocabulary test for German-speaking adults. We addressed this gap by introducing the GAudI, a receptive vocabulary test designed for German-speaking adults aged between 18 and 30 years. The GAudI is an adaptation of the DAIVT [6], an open-access receptive vocabulary test validated in Dutch-speaking samples of higher education students. The aims of the present study were (1) to report on the development of the GAudI and (2) to provide evidence for its validity by comparing its results with those of the DAIVT and assessing the relationships of the GAudI to other aspects of linguistic experience: print exposure, grammatical knowledge and productive vocabulary.

The data collected from 168 participants support the notion that the GAudI is a valid test of vocabulary size discriminating well between test takers. Participants’ performance on the GAudI was neither at ceiling nor at floor. In fact, the range (0.46 to 0.95) and standard deviation (SD = 0.12) suggest high variability in participant scores within the current sample. Item difficulty increased almost linearly (Fig 2), suggesting a good range of relatively easy to relatively difficult items. Furthermore, the reliability measure of Cronbach’s alpha suggests that the GAudI had great internal consistency (α=0.88). These reliability measures were almost identical to those of the DAIVT: recall that the DAIVT was tested on two samples, 49 Belgian first year university students aged between 17 and 22 years, and 108 Dutch speakers aged between 17 and 26 years, who were or had previously been in higher education. For both samples, Bousard and Brysbaert reported great internal consistency (first sample α=0.89, second sample α=0.88, [6]). Comparable to the GAudI, none of the DAIVT participants had a perfect score and no test item was responded to correctly by all participants. These measures demonstrate that the GAudI contains items with varying degrees of difficulty, eliciting high response variability in the current sample.

### Relationship between tests of linguistic experience

In order to relate the GAudI to other tests measuring linguistic experience, we used the German *Author Recognition Test* [53] as a measure of print expose, two antonym production tests measuring access to word meanings (as one aspect of productive vocabulary), and a book language grammar test measuring exposure to different grammatical categories. Additionally, we included the PPVT-DE to be able to relate the GAudI to another measure of receptive vocabulary, acknowledging that the test has been normed in adolescents but not adults. As predicted, all measures of linguistic experience were positively correlated with the GAudI, though the correlation of the timed version of the antonym production test did not reach statistical significance. The latter was most likely the case because the timed antonym production test was the only test for which the dependent variable was speed-based rather than accuracy-based. The strongest correlations were observed between GAudI, GART and the PPVT-DE, while moderate correlations were observed between the GAudI and the book language grammar and untimed antonym production tests, providing evidence for the notion that these tests tap into different facets of the same underlying construct (i.e., linguistic experience). We performed an exploratory factor analysis to determine how strongly the six tests loaded on a common factor. All variables except the timed antonym production test loaded significantly on the same factor. Both receptive vocabulary tests, GAudI (0.85) and PPVT-DE (0.86), had equally strong factor loadings followed by the GART (0.63), the untimed antonym production test (0.51), the book language grammar test (0.46), and the timed antonym production test (0.15). The factor loadings thus mirror the pattern of correlations between the GAudI and the other five tests.

As a measure of receptive vocabulary, the GAudI correlated strongly with the PPVT-DE. Both tests also had the highest and similar factor loadings, which reflect their similarity in task demands and type of knowledge required. Since both tests were designed to measure receptive vocabulary and in fact use the same test design, one might have expected an even stronger correlation. However, the strong left-skew of the PPVT-DE (Table 1) indicates that the PPVT-DE was indeed too easy for the current sample. In spite of this caveat, the correlation between the two tests was remarkably high (r = 0.73). To put this in perspective, we compared the correlation between GAudI and PPVT-DE in the current study with the correlation reported for the DAIVT and PPVT-NL in [6]. For both, the Belgian university student sample (r = 0.77, p < .001) and the second Dutch speaker sample (r = 0.76, p < .001), the DAIVT correlated only slightly more strongly with the PPVT-NL than the GAudI did with the PPVT-DE. The small difference might have arisen due to the PPVT-DE being normed in children and adolescents with a maximum age of 16 years, while the PPVT-NL was also normed in adults [40].

As a measure of print exposure, good performance on the GART relates to ample exposure to written texts and thus higher probability of knowing author names [21,22]. The high factor loading of the GART (0.63) together with its strong correlation to the GAudI and PPVT-DE suggest a positive relationship between author knowledge and vocabulary size. Presumably both, a rich vocabulary and author knowledge, stem from engagement with written language [21–23,25,70]. As explained in the introduction, written texts feature more infrequent words than spoken language. The correlation between the receptive vocabulary tests and GART might thus be explained by frequent reading benefitting both the acquisition of high- and low-frequency words and the acquisition of author knowledge.

The newly developed tests, i.e., both antonym production tests and the book language grammar test, had weaker loadings on the linguistic experience factor and weaker correlations with the GAudI. However, the factor loadings for the untimed antonym production test (0.51) and book language grammar test (0.46) together with their moderate correlations to the GAudI support the notion that these tests still contribute significantly to the underlying construct of linguistic experience, while also measuring different skills that may not be directly related to linguistic experience. In the untimed antonym production test, participants must access the conceptual representation of spoken words (varying in word frequency) in order to prepare their *production* of the antonym. In the GAudI, participants must also access conceptual knowledge of the spoken target words to be able to map its semantics on the semantics extracted from the image response alternatives, but *without* an additional word production process. The weak loadings and lack of statistical significance of the correlations for the timed antonym production test indicate that this test did not share many underlying processes with the GAudI. This could mean that performance on the timed antonym production test might have been primarily determined by domain-general processing speed, rather than language-related experience.

Further evidence for shared variance across tests of linguistic experience can be gleaned from the correlation between the GAudI and the book language grammar test. These tests tap different types of linguistic experience, as indicated by the relatively weak factor loading for the grammar test. Whereas the GAudI taps semantic representations at the word level (word knowledge), the book language grammar test taps experience with different grammatical constructions, and mapping the experience onto the encountered sentential input. The weak positive correlation between these tests may be taken to indicate that the GAudI and grammar test share (some) underlying components, but display different types of linguistic experience. Regardless of this shared variance, the comparatively weaker factor loading and correlation implies that the book language grammar test might be a less ideal measure of linguistic experience, considering that it has not been previously validated.

As expected, the internal consistency was great to excellent for the two existing tests, i.e., the PPVT-DE (α=0.8) and the GART (α=0.94). This was also the case for the timed antonym production test (α=0.92). However, it was comparatively low for the book language grammar test (α=0.68) and untimed antonym production test (α=0.62). While the alpha of 0.68 may be considered close to acceptable [71], the alpha of 0.62 for the untimed antonym production test stems most likely from the small number of items, which often leads to lower internal consistency levels. Both tests were adapted from already existing ones. Compared to the original antonym production test (α=0.48) [54], the reliability for the untimed antonym production test was improved in the current study. For the grammar test, reliability was comparable between the Dutch (α=0.65) [54] and the German version (α=0.68). This may be taken as support for the argument that translation of existing tests is an efficient tool for test development [42], while keeping in mind that the untimed antonym production test and book language grammar test are newly developed and have comparatively small item sets.

### Limitations and recommendations for use of the GAudI

We introduce a new test of receptive vocabulary for German-speaking adults aged between 18 and 30. Its picture-based format, where word meanings have to be mapped onto pictures, complements other tests with different formats, such as the *Receptive German 3 Vocabulary Size Test* [30] or Schmidt and Metzler’s *Wortschatztest* [36], whose formats are based on written word forms. There are three points that have to be considered when using the GAudI.

First, when assessing vocabulary, it is advisable to use more than one measure of vocabulary to account for effects of the test format and selection of stimuli [6]. While the GAudI can be one such test, we recommend complementing it with another measure of vocabulary knowledge. Additionally, as a test of receptive vocabulary, the GAudI assesses *one* aspect of linguistic experience. While some studies have used vocabulary size as a proxy for linguistic experience [72,73], vocabulary size alone is not sufficient to capture the breadth of an individual’s experience with a language. Other tests are needed to assess linguistic experience and skills comprehensively. This might include author recognition tests as a measure of print exposure, grammar tests to capture knowledge of complex syntactic constructions, and productive vocabulary tests. These tests capture unique facets of a person’s experience in comprehending and producing language. Though the results of this study support the notion that all of the tests measure different aspects of linguistic experience, we do not claim to measure linguistic experience exhaustively with these tests. For a more complete picture of linguistic experience, one might consider including measures such as reading comprehension or spelling abilities as well.

Second, vocabulary may also be considered a more general measure of an individual’s knowledge, rather than linguistic experience alone. Vermeiren et al. [74] recently argued that vocabulary and author knowledge may both be considered measures of crystallized intelligence, which is (cultural) knowledge that is stored in long-term memory [75,76]. The strong relationship between vocabulary and author knowledge arises because both belong to the kind of knowledge that is entrenched in memory. Thus, word knowledge can be seen as a component of crystallized intelligence and so it has been proposed that tests of crystallized intelligence should include vocabulary tests [42,74]. The relationship between linguistic experience and the broader trait of crystallized intelligence needs to be examined in further work.

Lastly, the results reported here are based on a sample of 168 German-speaking adults aged between 18 and 30 years. The majority of these participants were female (n = 132) and/or students in higher education (n = 134). Our participants are thus WEIRD (Western, Educated, Industrialized, Rich, and Democratic [77]). The results may differ in samples from more diverse educational backgrounds. However, given how often university students participate in psychological research, we consider the GAudI a useful research tool and hope it will be widely used. It should be kept in mind that the GAudI was developed for research purposes only and is, thus, not recommended as a tool for clinical use.

## Conclusion

Despite the wide range of potential applications, there is currently no standardized receptive vocabulary test available for German-speaking adults. Most available tests were designed for assessment in children or foreign language learners. In the present study, we addressed this gap and introduced the *German Auditory and Image Vocabulary Test* (GAudI). The GAudI is a free receptive vocabulary test that has been validated in a sample of 168 German-speaking adults aged between 18 and 30 years. The test uses a multiple-choice format where participants are instructed to select target images corresponding to spoken words. Our results indicate that the test is a reliable tool. Performance on the test correlates well with tests measuring different facets of linguistic experience, including receptive and productive vocabulary, book language grammar and print exposure. The GAudI is freely available online for research purposes at https://ems13.mpi.nl/bq4_customizable_de/researchers_welcome.php.

## Supporting information

S1 Table Trial structure of the GAudI including target and distractor images. Images marked in green are the targets. Images reprinted from [6] under a CC BY license, with permission from Marc Brysbaert, original copyright 2021.
(PDF)

S2 Table Item set of the GAudI. Frequency designations: Zipf_Freq =  Zipf frequency obtained from SUBTLEX-DE [49]; HK_Leipzig =  *Häufigkeitsklassen* (frequency classes) obtained from *Projekt Deutscher Wortschatz* (Project German Vocabulary) of Leipzig University [51]; dwds_freq =  frequency level obtained from *Digitales Wörterbuch der deutschen Sprache* (DWDS) [52]; dwds_hits =  number of tokens in the DWDS corpus [52].
(PDF)

S3 Table Item set of the antonym production test (untimed). Frequency designations for cues and targets: Zipf_freq =  Zipf frequency obtained from SUBTLEX-DE [49]; HK_Leipzig =  *Häufigkeitsklassen* (frequency classes) obtained from *Projekt Deutscher Wortschatz* (Project German Vocabulary) of Leipzig University [51]; dwds_freq =  frequency level obtained from *Digitales Wörterbuch der deutschen Sprache* (DWDS) [52].
(PDF)

S4 Table Item set of the antonym production test (timed). Frequency designations for cues and targets: Zipf_freq =  Zipf frequency obtained from SUBTLEX-DE [49]; HK_Leipzig =  *Häufigkeitsklassen* (frequency classes) obtained from *Projekt Deutscher Wortschatz* (Project German Vocabulary) of Leipzig University [51]; dwds_freq =  frequency level obtained from *Digitales Wörterbuch der deutschen Sprache* (DWDS) [52].
(PDF)

S5 Table Item set of the book language grammar test. Critical parts of the sentence are marked in bold print. Asterisks mark grammatically incorrect sentences.
(PDF)

S6 Table Output of the Rasch analysis on the item set of the GAudI. Infit and outfit mean square values between 0.5 and 1.5 are considered productive for measurement, while values below 0.5 are deemed less productive, but not degrading.
(PDF)

S7 Fig Item Trace Plot for the GAudI. Item characteristics curves for each item in the GAudI.
(TIF)
