# Supplementary material for: The German Auditory and Image (GAudI) vocabulary test: A new German receptive vocabulary test and its relationships to other tests measuring linguistic experience
Source: PLoS One. 2025 Apr 28;20(4):e0318115. doi: 10.1371/journal.pone.0318115 (PMC12036901; doi:10.1371/journal.pone.0318115)
Supplement: S1 Table — Images marked in green are the targets. Images reprinted from [6] under a CC BY license, with permission from Marc Brysbaert, original copyright 2021. (PDF) [file pone.0318115.s001.pdf]

Practice item: Stuhl			
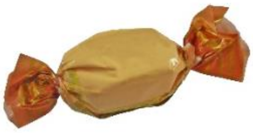	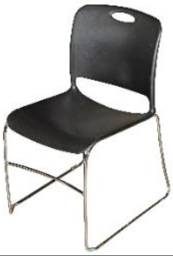	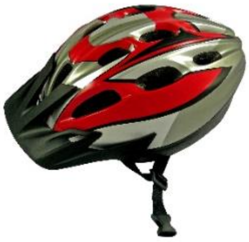	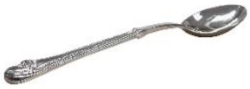
1	2	3	4

1. Barett			
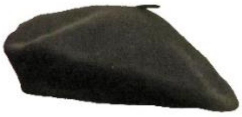	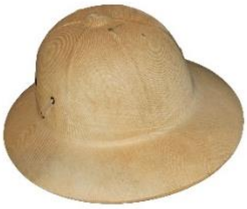	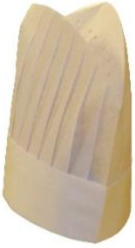	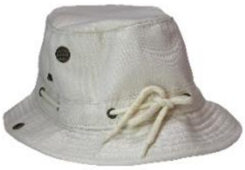
1	2	3	4

2. Fassade			
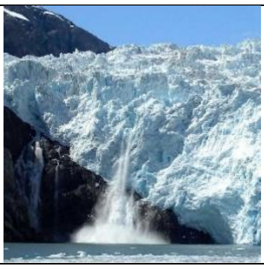	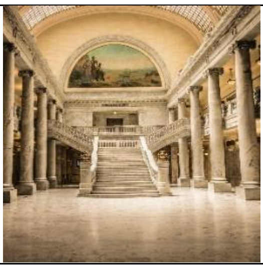	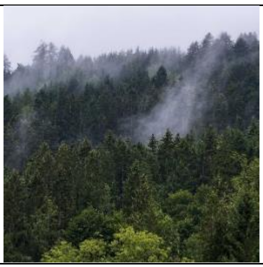	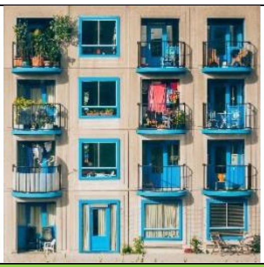
1	2	3	4

3. Ametropie			
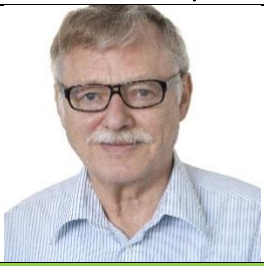	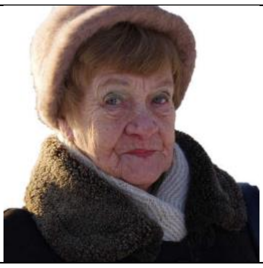	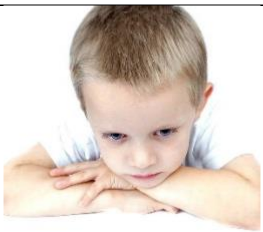	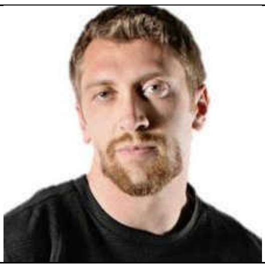
1	2	3	4

4. Kormoran			
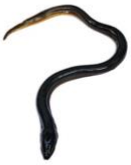	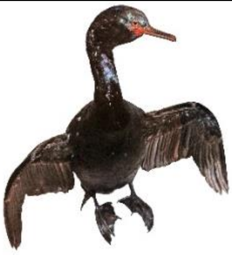	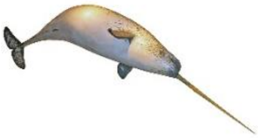	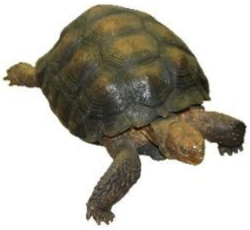
1	2	3	4

5. biwakieren			
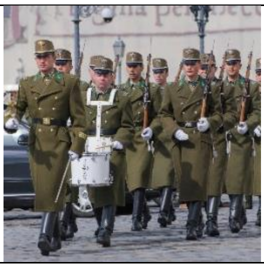	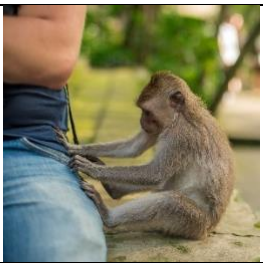	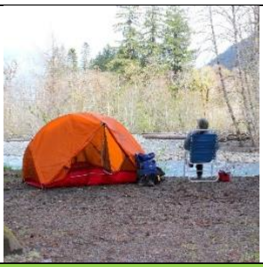	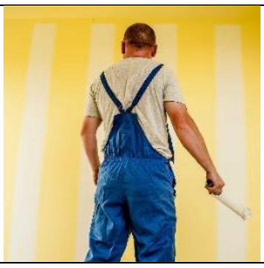
1	2	3	4

6. ekstatisch			
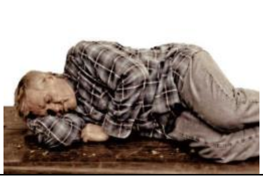	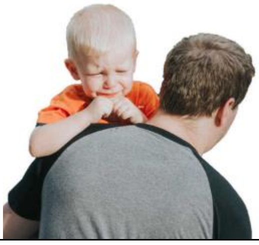	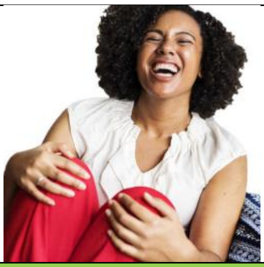	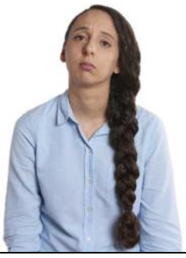
1	2	3	4

7. konzentrisch			
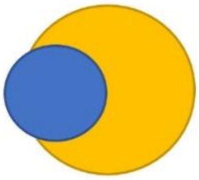	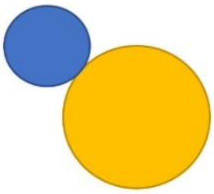	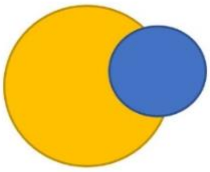	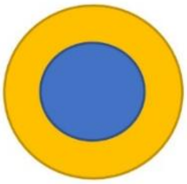
1	2	3	4

8. Triade			
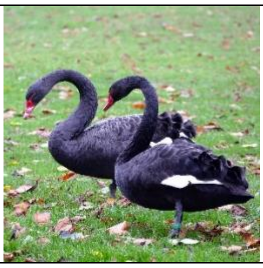	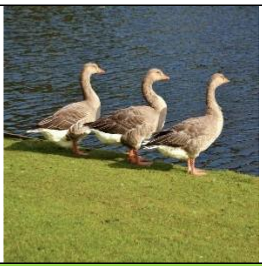	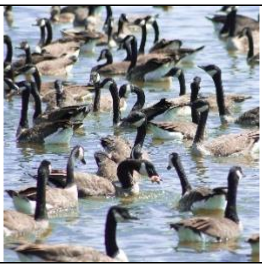	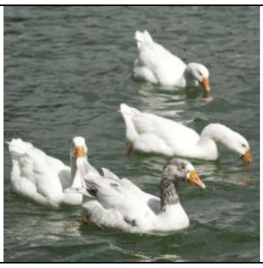
1	2	3	4

9. Grummet			
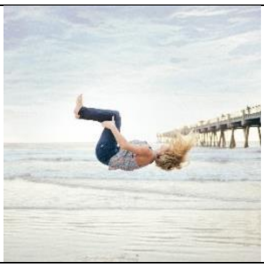	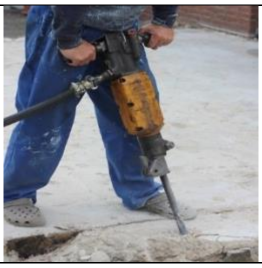	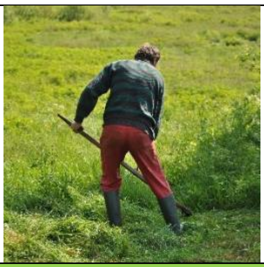	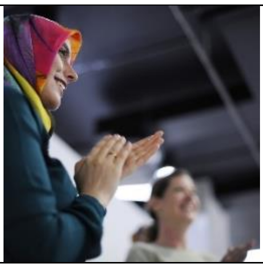
1	2	3	4

10. Wirbel			
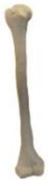	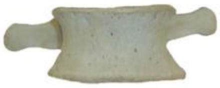	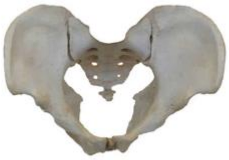	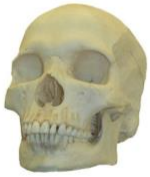
1	2	3	4

11. Bolide			
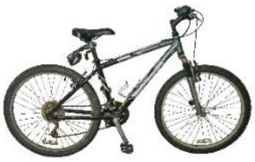	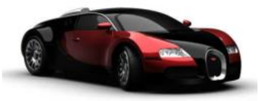	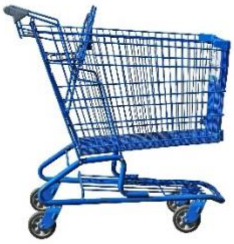	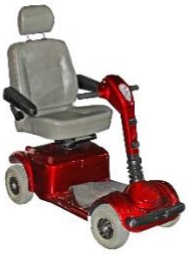
1	2	3	4

12. Paläontologe			
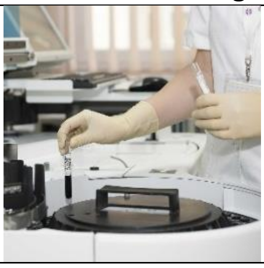	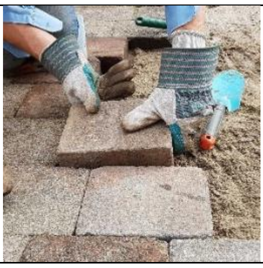	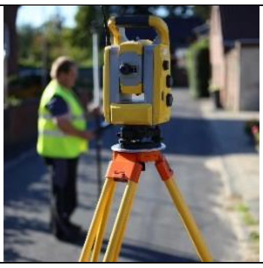	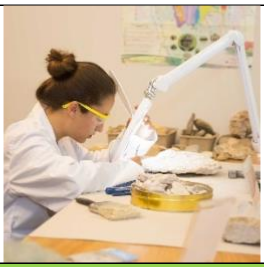
1	2	3	4

13. gelappt			
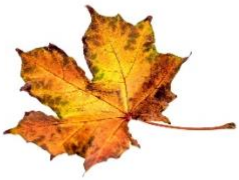	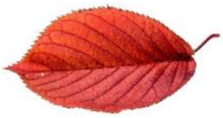	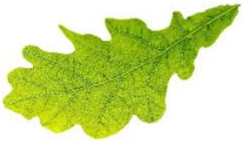	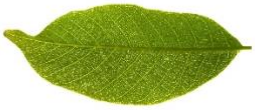
1	2	3	4

14. prokrastinieren			
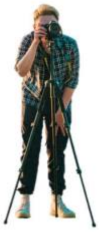	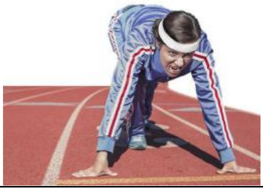	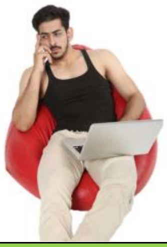	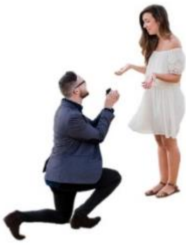
1	2	3	4

15. Organigramm			
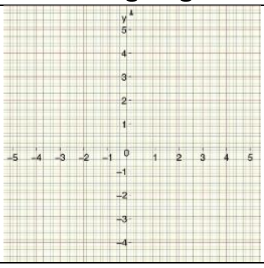	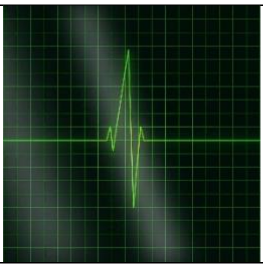	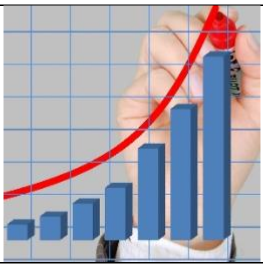	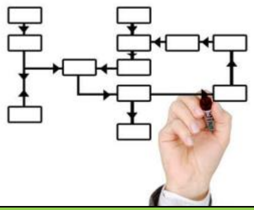
1	2	3	4

16. kandieren			
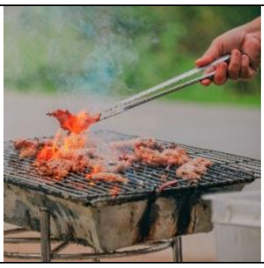	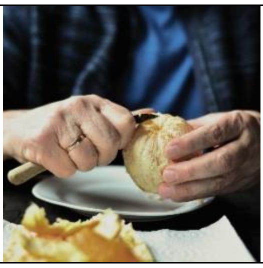	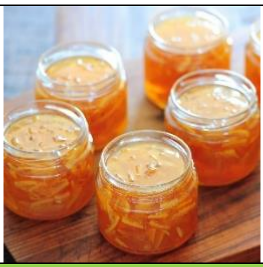	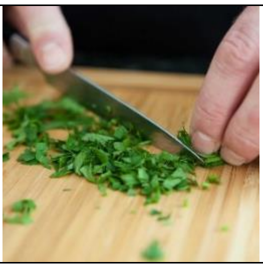
1	2	3	4

17. apportieren			
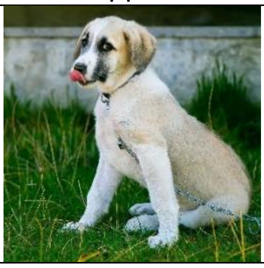	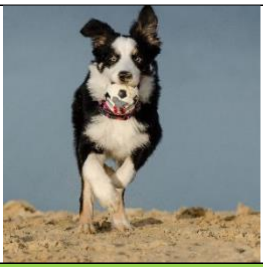	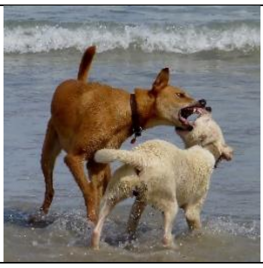	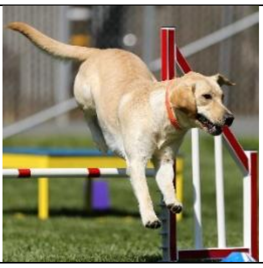
1	2	3	4

18. Emission			
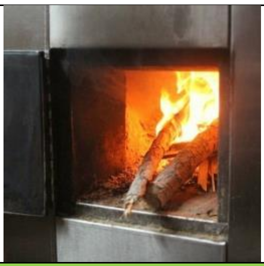	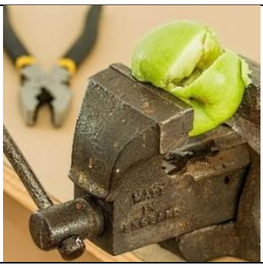	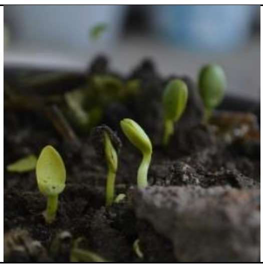	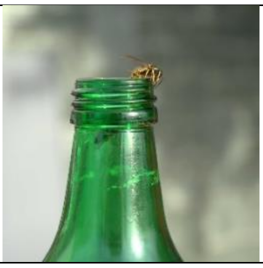
1	2	3	4

19. Spachtel			
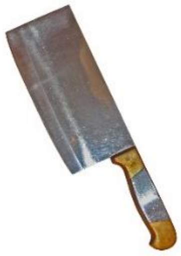	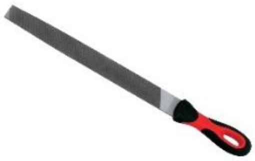	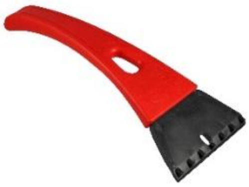	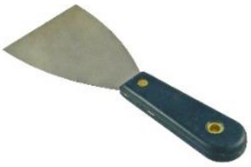
1	2	3	4

20. opulent			
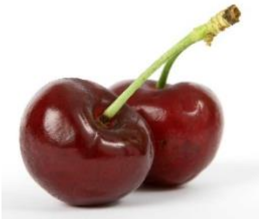	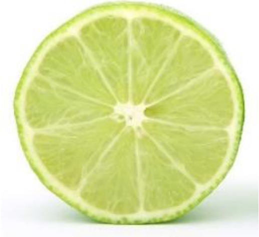	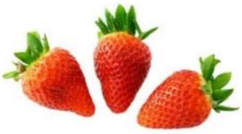	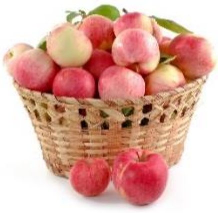
1	2	3	4

21. Metronom			
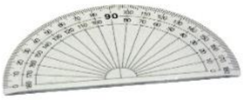	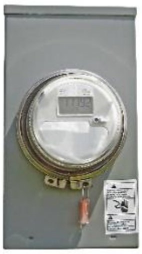	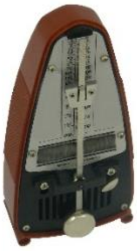	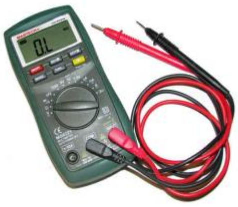
1	2	3	4

22. Schwaden			
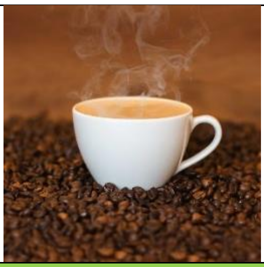	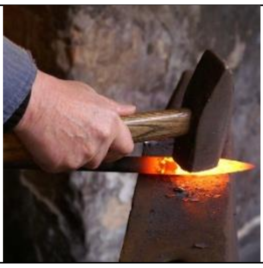	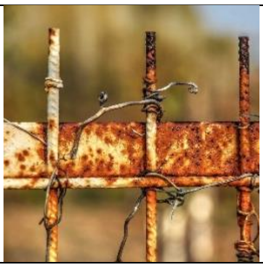	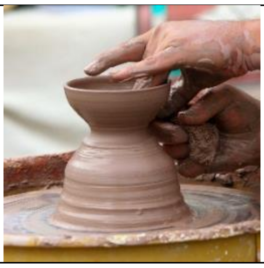
1	2	3	4

23. Pistolengriff			
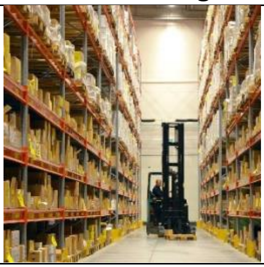	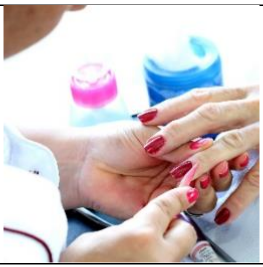	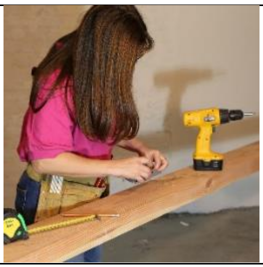	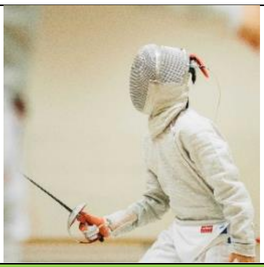
1	2	3	4

24. Habit			
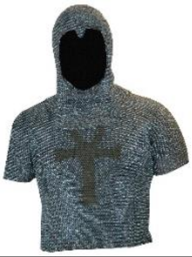	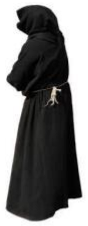	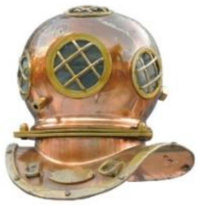	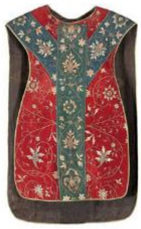
1	2	3	4

25. Perkussion			
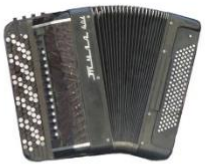	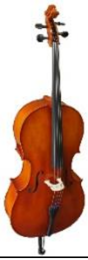	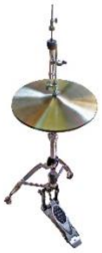	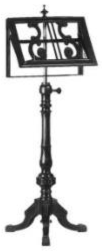
1	2	3	4

26. degustieren			
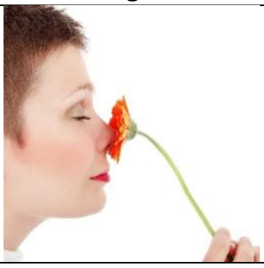	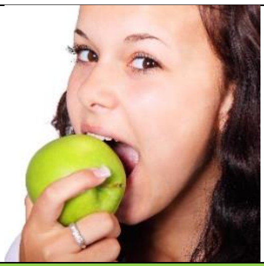	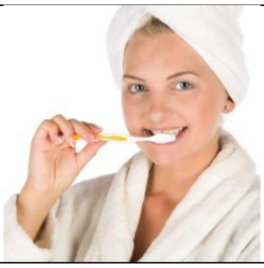	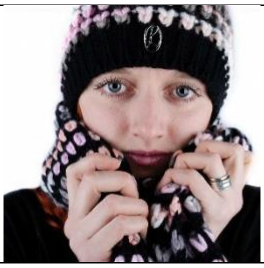
1	2	3	4

27. urban			
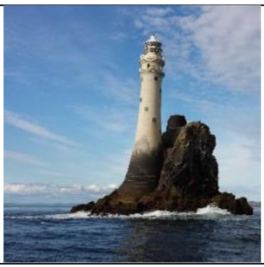	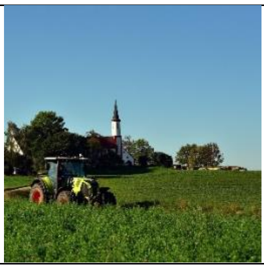	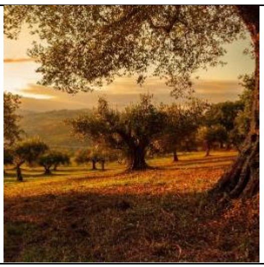	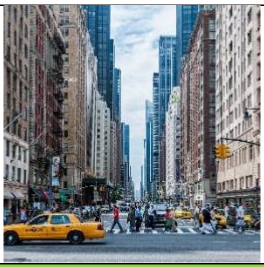
1	2	3	4

28. Philatelie			
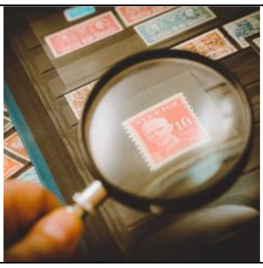	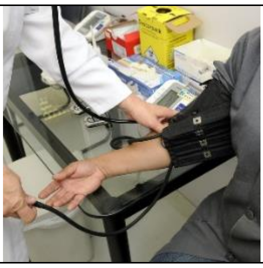	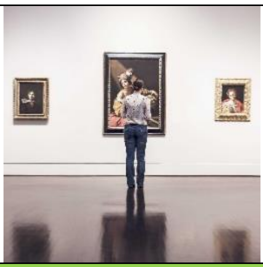	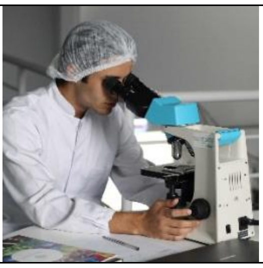
1	2	3	4

29. seihen			
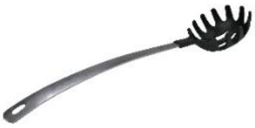	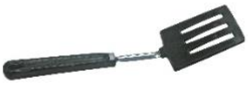	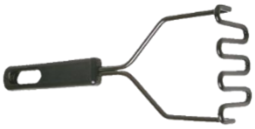	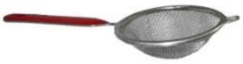
1	2	3	4

30. Galionsfigur			
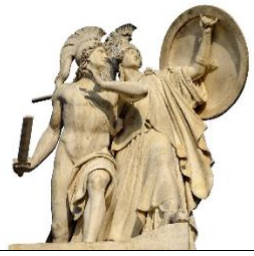	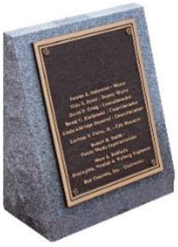	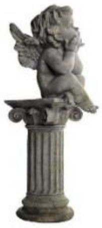	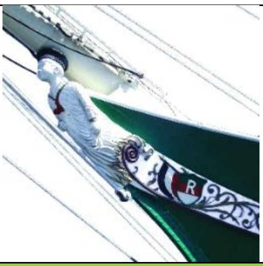
1	2	3	4

31. gastronomisch			
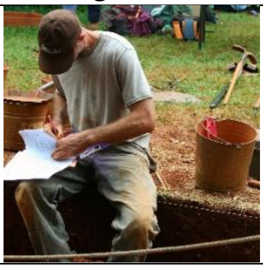	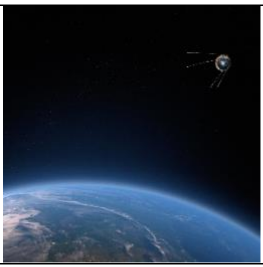	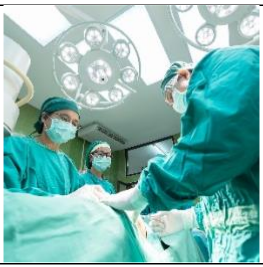	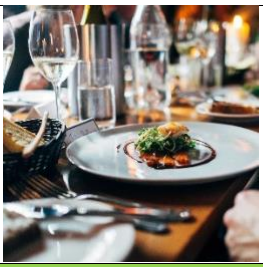
1	2	3	4

32. palpieren			
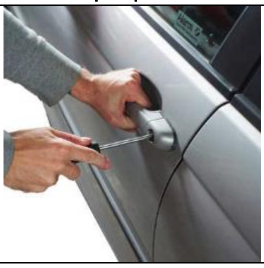	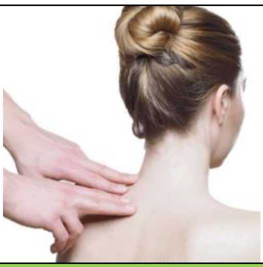	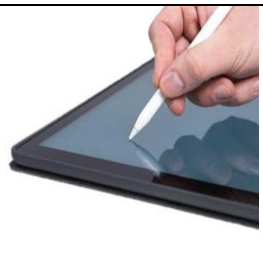	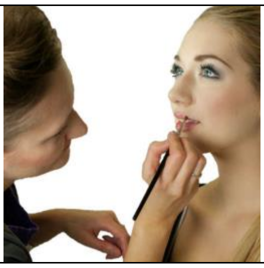
1	2	3	4

33. Arkade			
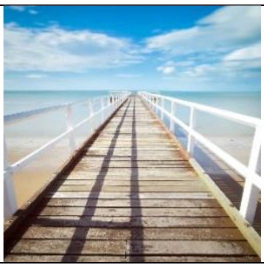	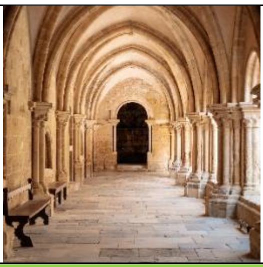	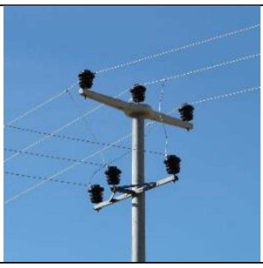	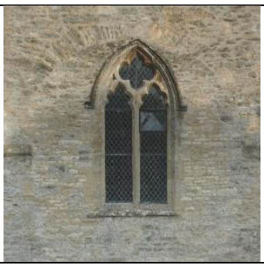
1	2	3	4

34. Artefakt			
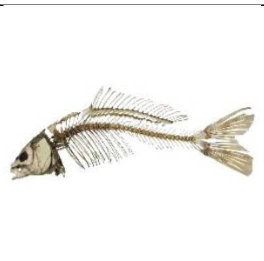	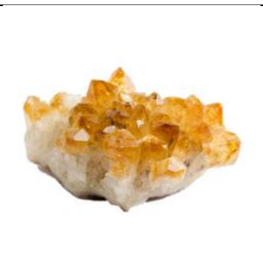	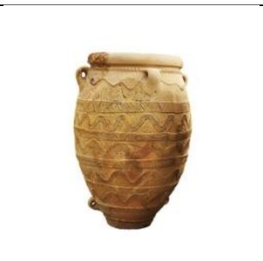	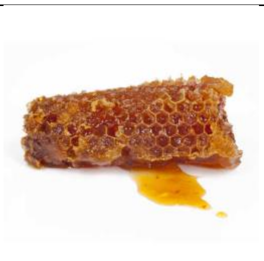
1	2	3	4

35. posterior			
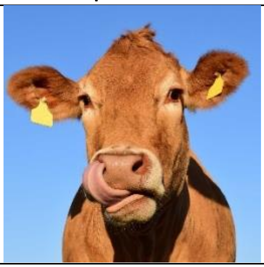	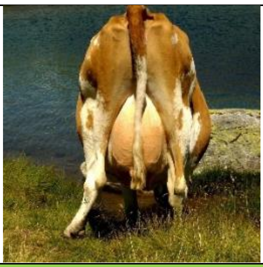	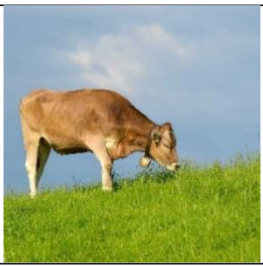	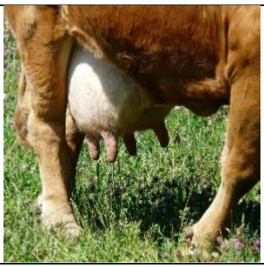
1	2	3	4

36. Hellebarde			
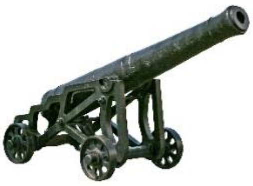	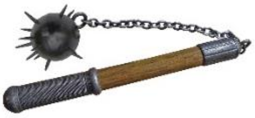	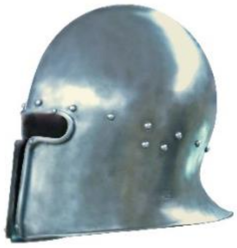	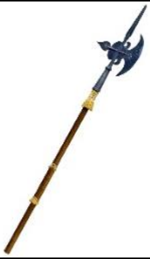
1	2	3	4

37. klandestin			
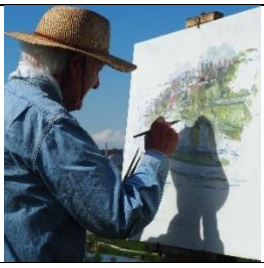	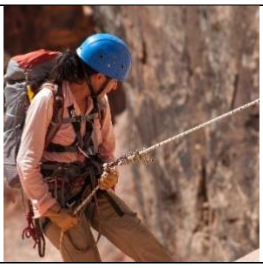	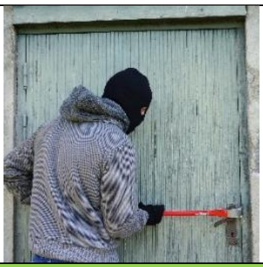	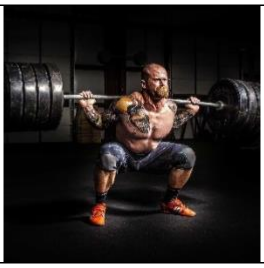
1	2	3	4

38. lanzettförmig			
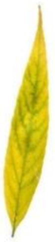	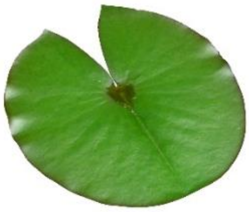	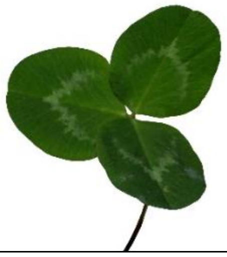	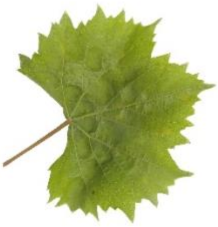
1	2	3	4

39. sinnieren			
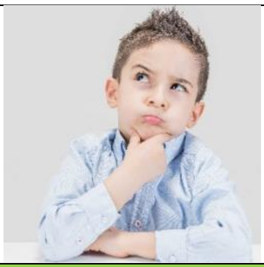	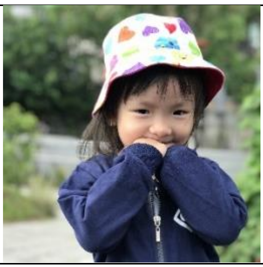	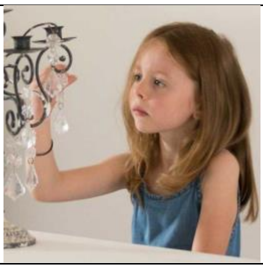	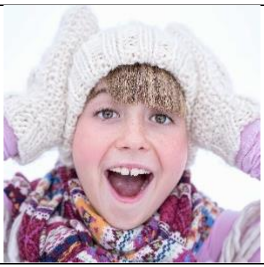
1	2	3	4

40. Rosette			
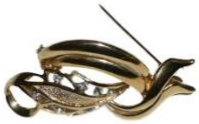	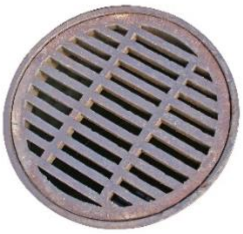	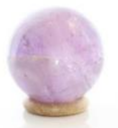	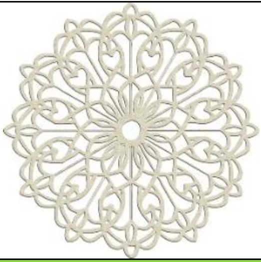
1	2	3	4

41. verschmähen			
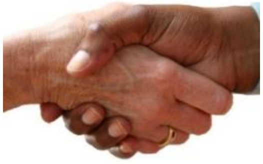	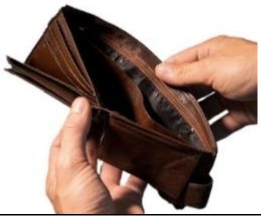	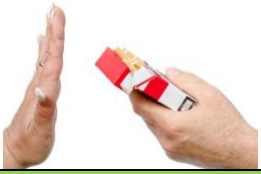	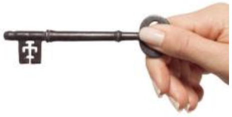
1	2	3	4

42. kumulieren			
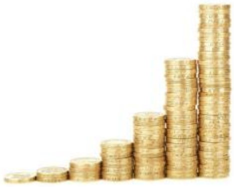	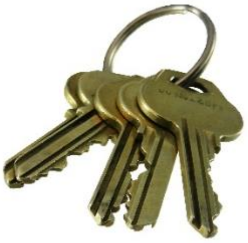	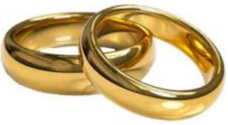	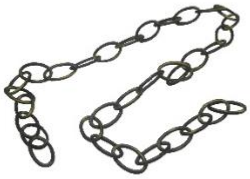
1	2	3	4

43. Wehr			
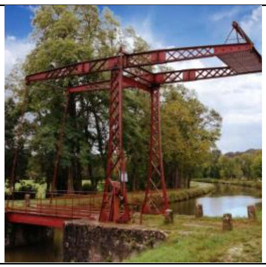	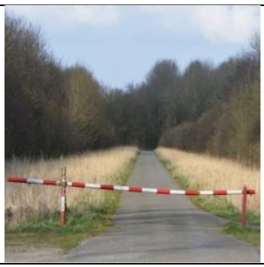	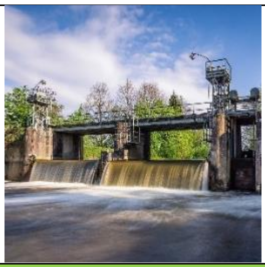	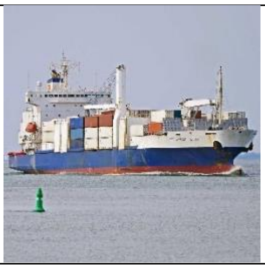
1	2	3	4

44. Gefälle			
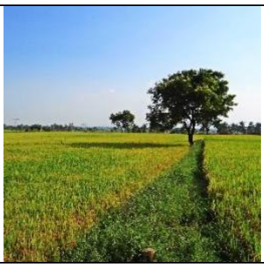	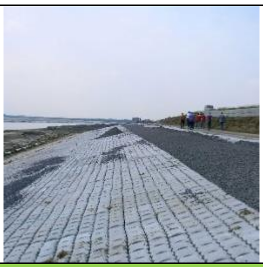	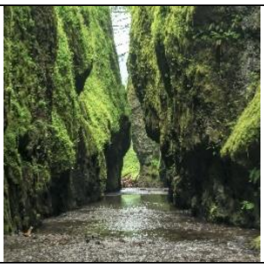	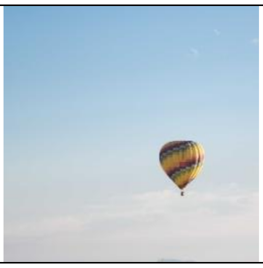
1	2	3	4

45. konferieren			
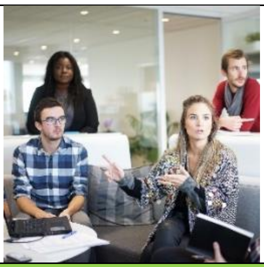	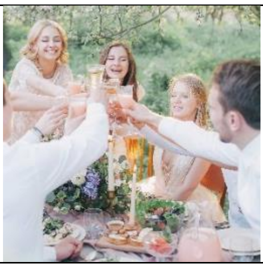	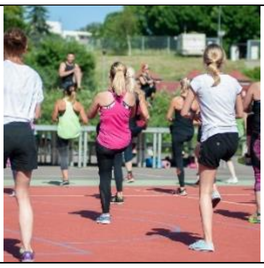	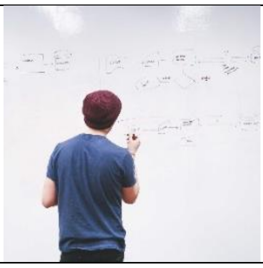
1	2	3	4

46. Aversion			
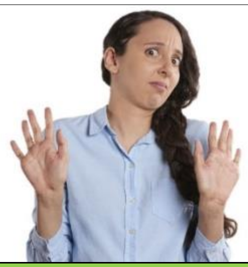	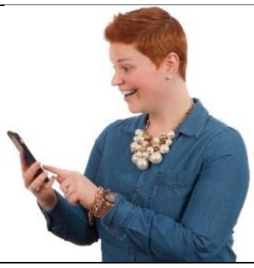	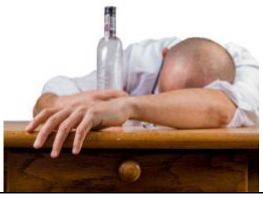	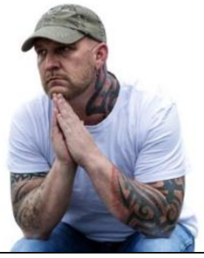
1	2	3	4

47. Büste			
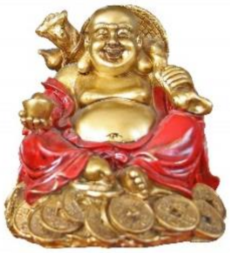	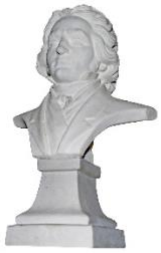	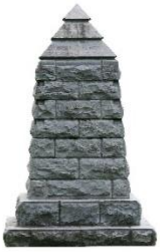	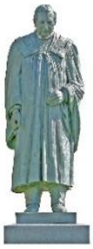
1	2	3	4

48. Neonat			
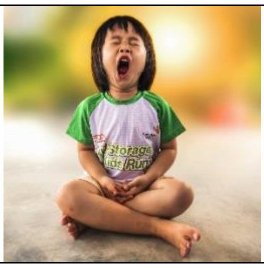	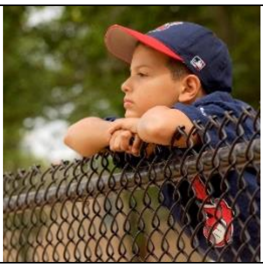	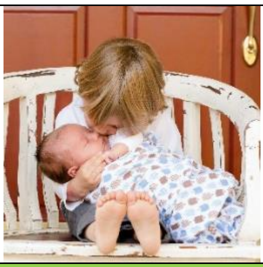	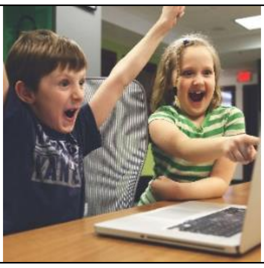
1	2	3	4

49. Pagode			
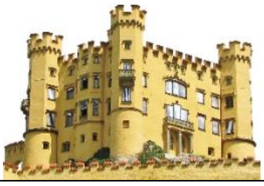	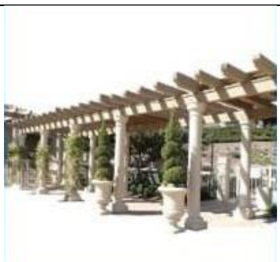	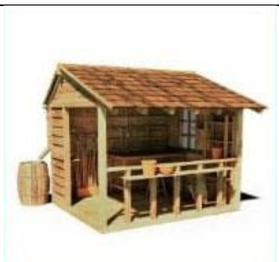	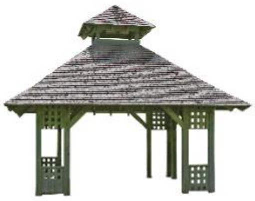
1	2	3	4

50. Ampere			
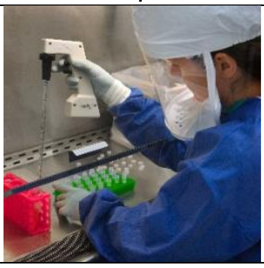	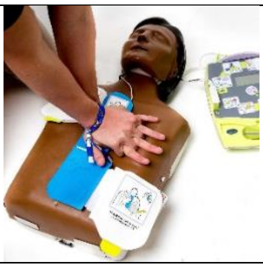	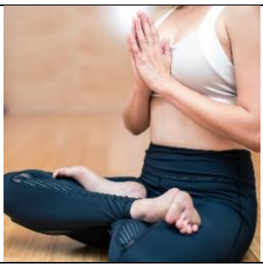	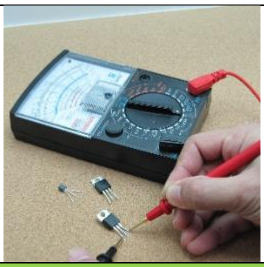
1	2	3	4

51. Halm			
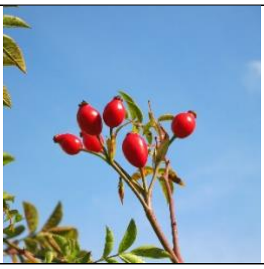	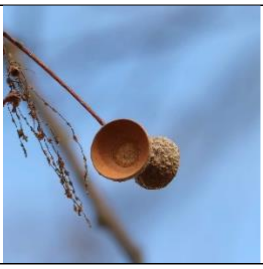	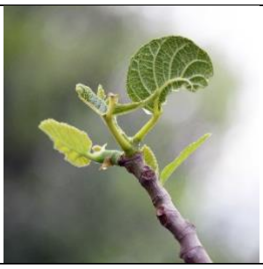	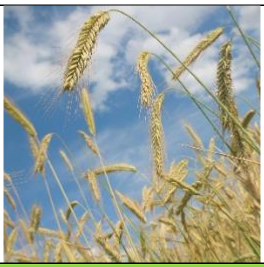
1	2	3	4

52. konisch			
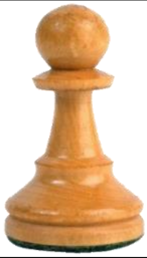	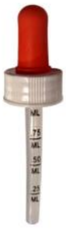	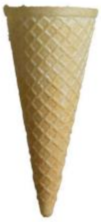	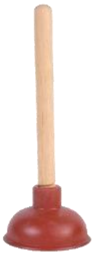
1	2	3	4

53. Feuchtgebiet			
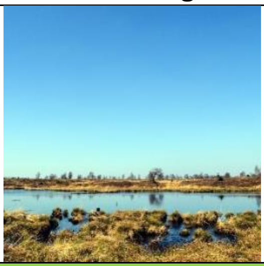	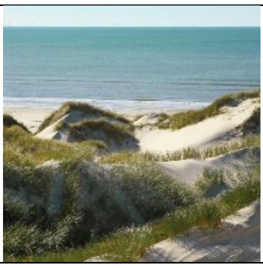	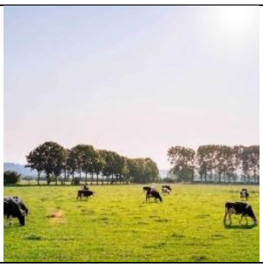	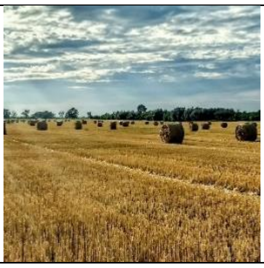
1	2	3	4

54. invertebrata			
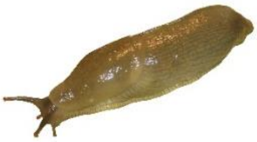	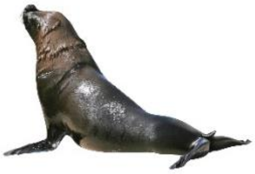	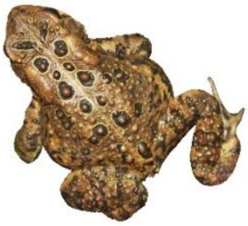	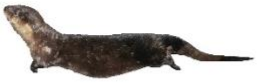
1	2	3	4

55. Erker			
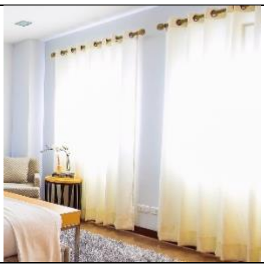	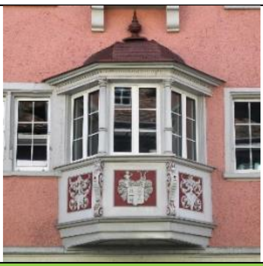	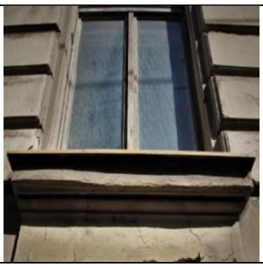	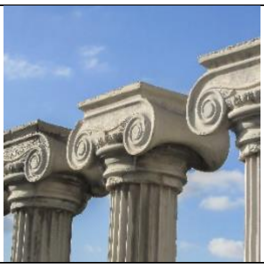
1	2	3	4

56. Konfekt			
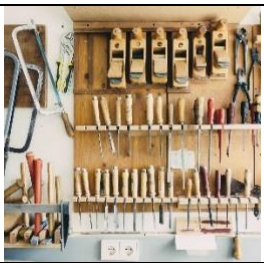	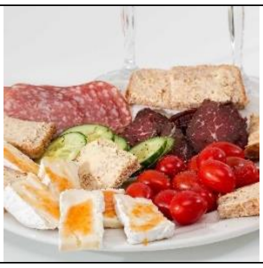	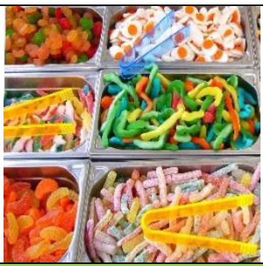	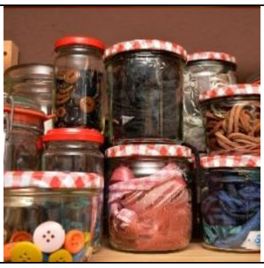
1	2	3	4

57. laben			
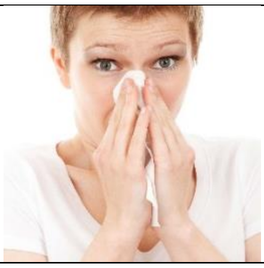	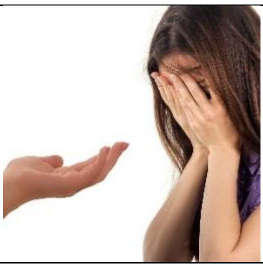	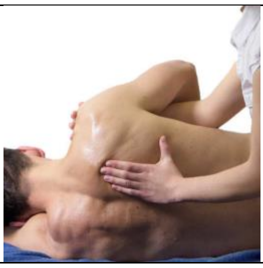	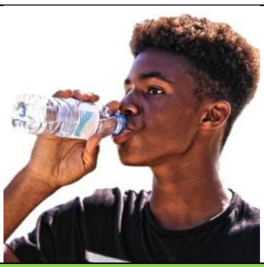
1	2	3	4

58. Degression			
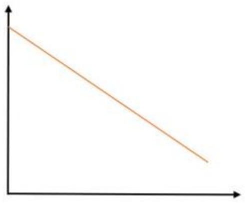	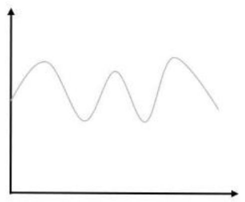	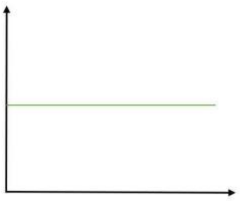	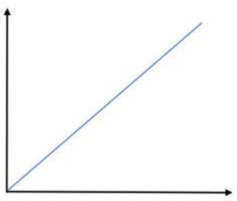
1	2	3	4

59. Safran			
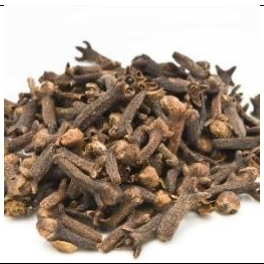	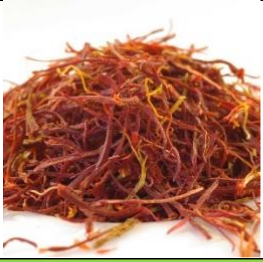	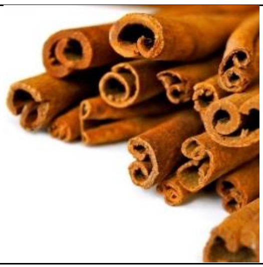	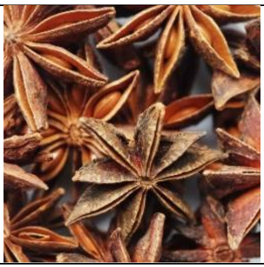
1	2	3	4

60. prekär

1	2	3	4
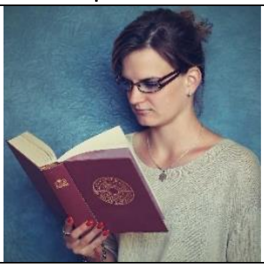	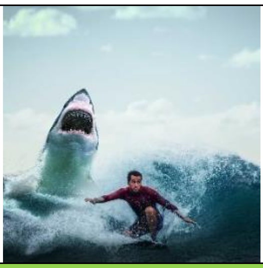	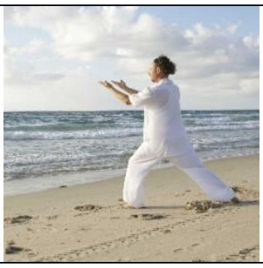	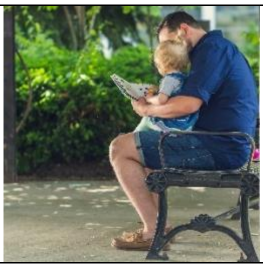

61. Mammologie

1	2	3	4
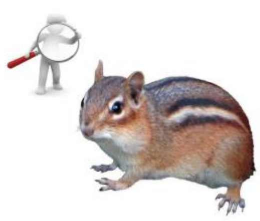	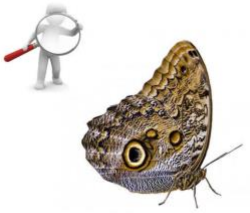	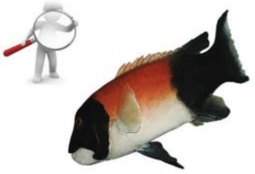	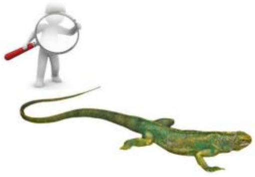

62. Zenturio

1	2	3	4
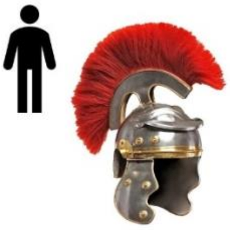	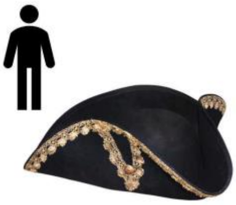	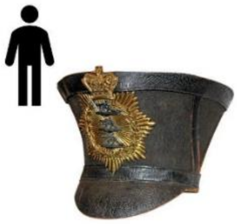	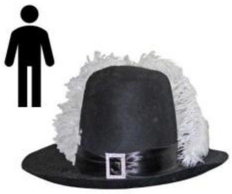

63. Kolonie

1	2	3	4
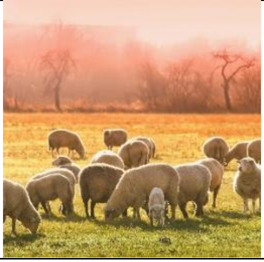	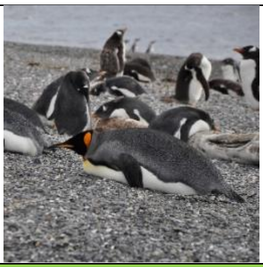	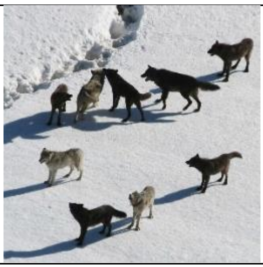	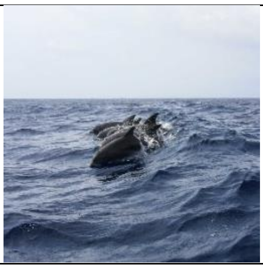

64. Konvoi			
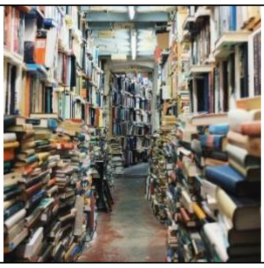	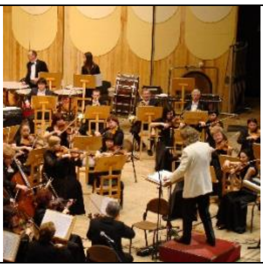	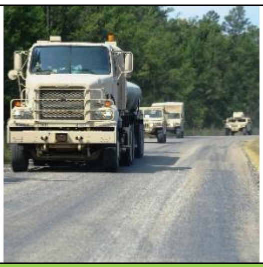	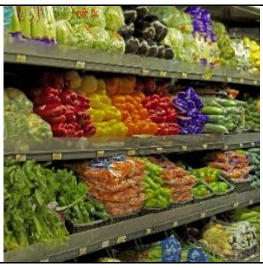
1	2	3	4

65. baufällig			
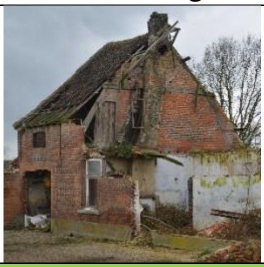	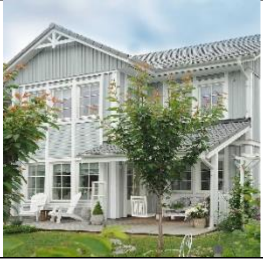	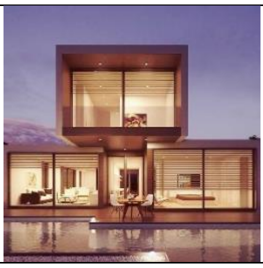	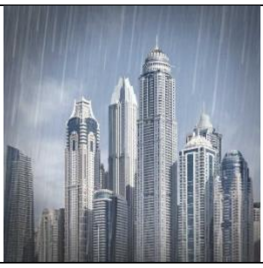
1	2	3	4

66. Kazoo			
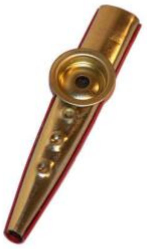	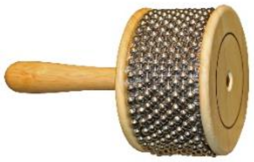	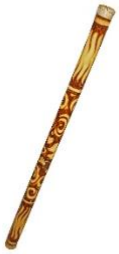	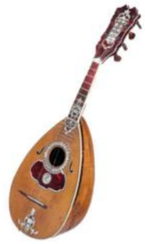
1	2	3	4

67. treuhänderisch			
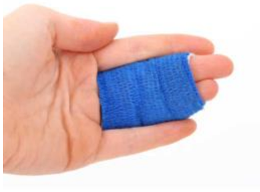	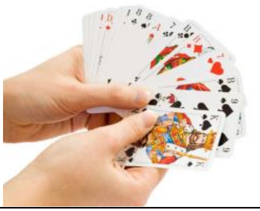	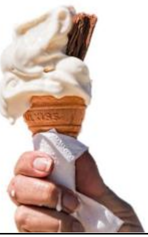	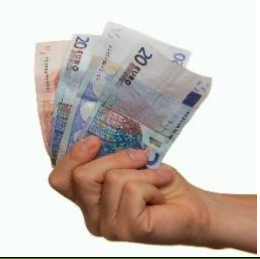
1	2	3	4

68. Bimsstein			
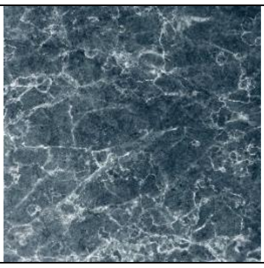	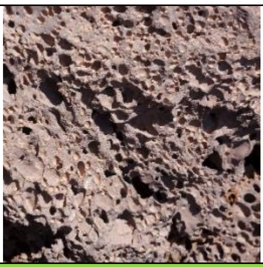	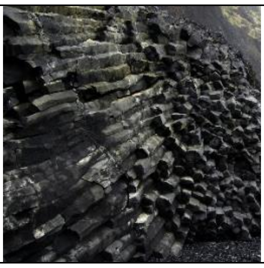	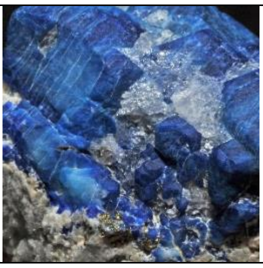
1	2	3	4

69. Eruption			
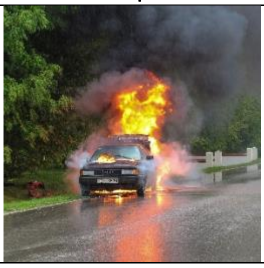	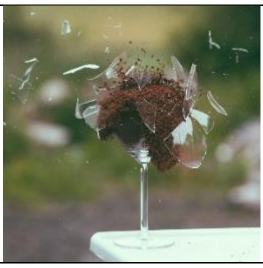	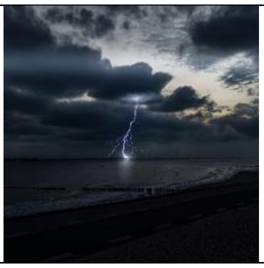	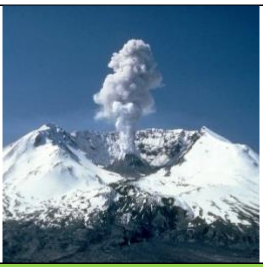
1	2	3	4

70. Couturier			
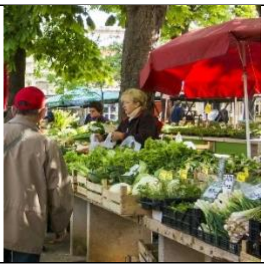	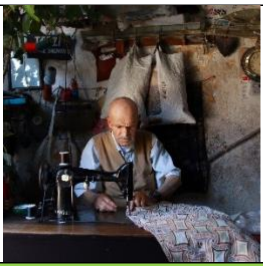	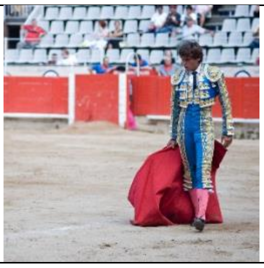	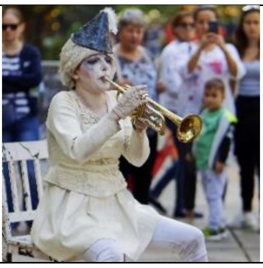
1	2	3	4

71. indigniert			
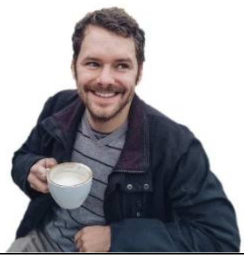	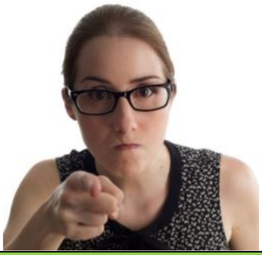	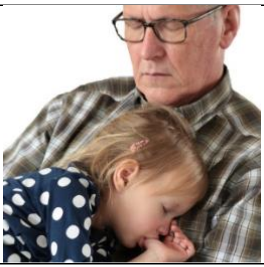	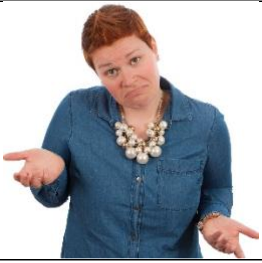
1	2	3	4

72. geothermal			
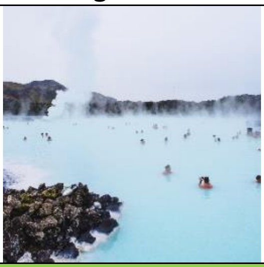	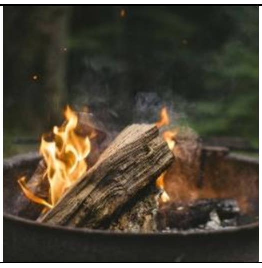	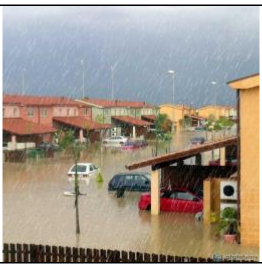	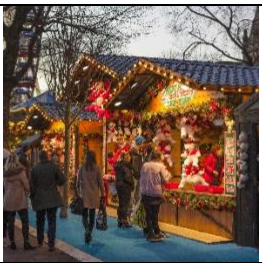
1	2	3	4

73. verbarrikadiert			
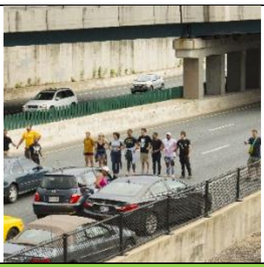	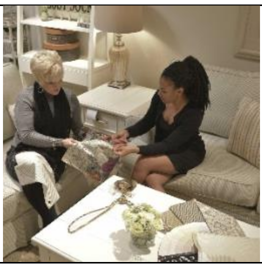	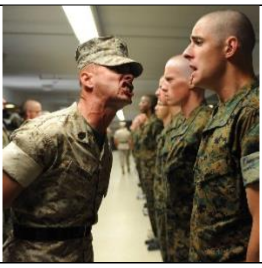	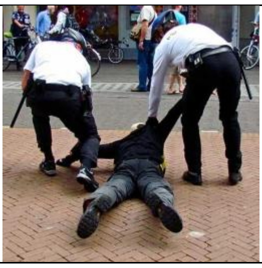
1	2	3	4

74. dressieren			
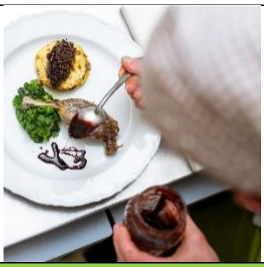	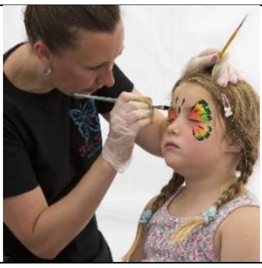	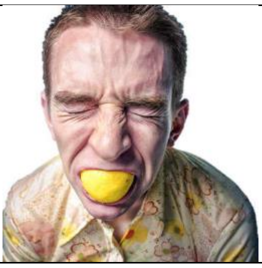	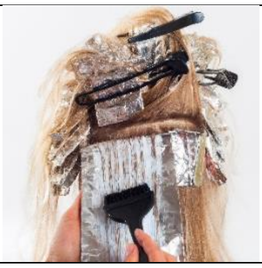
1	2	3	4

75. Diwan			
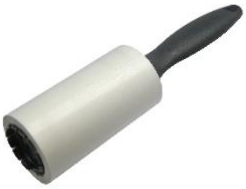	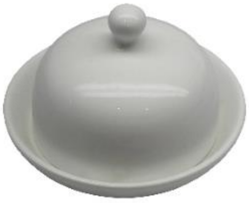	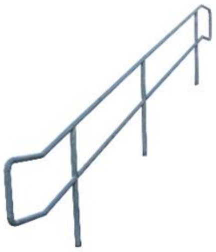	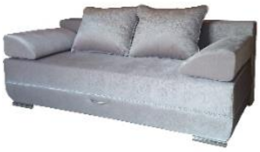
1	2	3	4

76. schelmisch

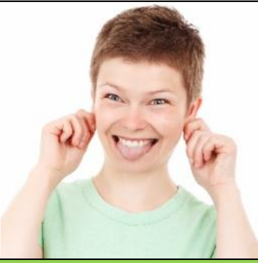	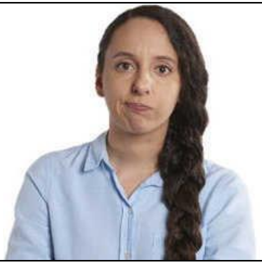	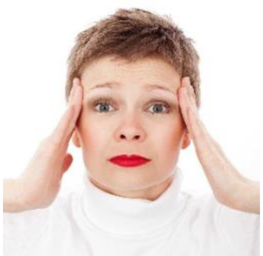	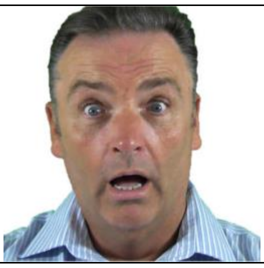
1	2	3	4

77. Piaffe

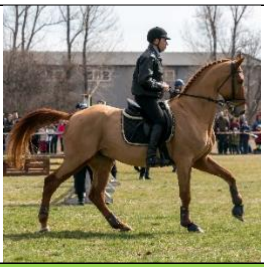	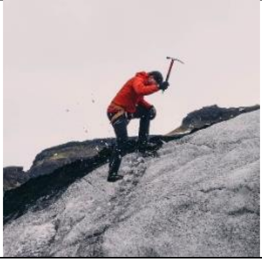	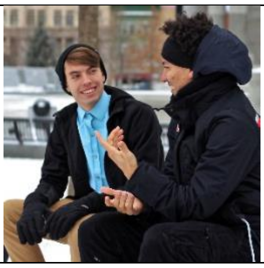	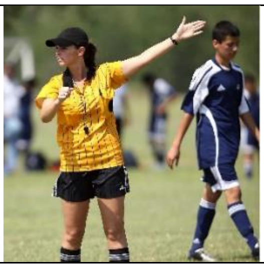
1	2	3	4

78. pektoral

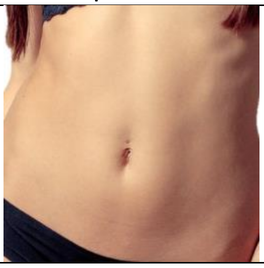	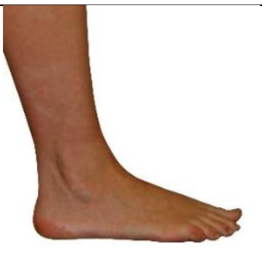	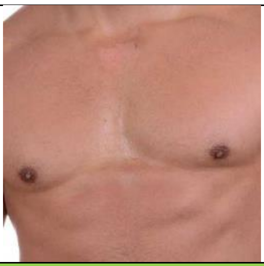	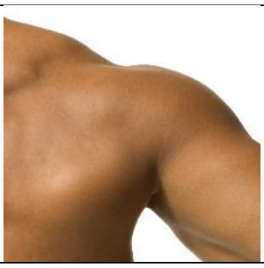
1	2	3	4

79. Bukett

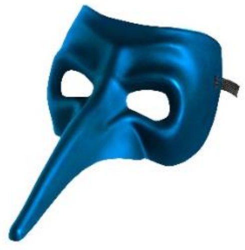	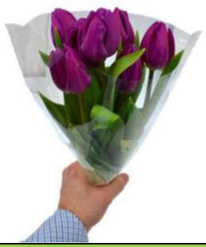	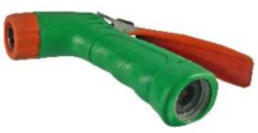	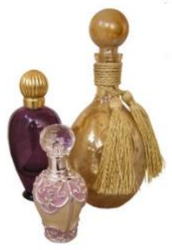
1	2	3	4

80. Irrigation			
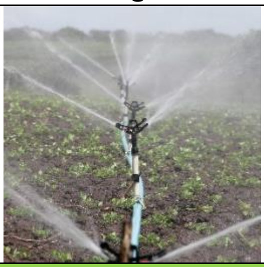	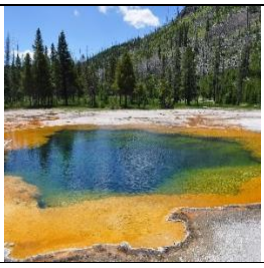	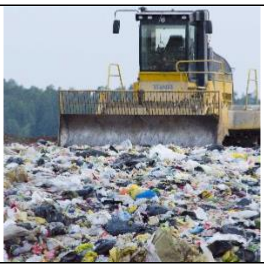	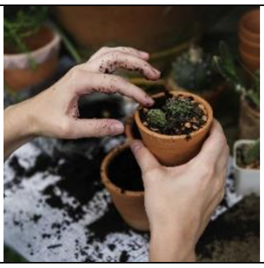
1	2	3	4

81. Tilde			
~	˘	¨	^
1	2	3	4

82. Animosität			
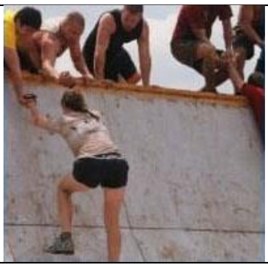	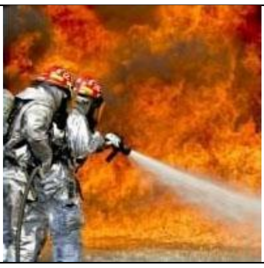	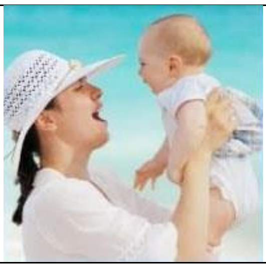	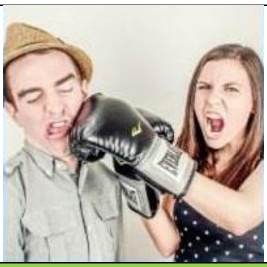
1	2	3	4

83. Sublimation			
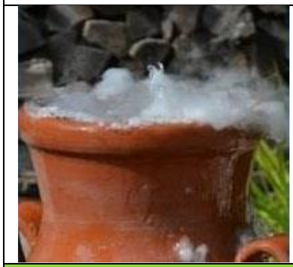	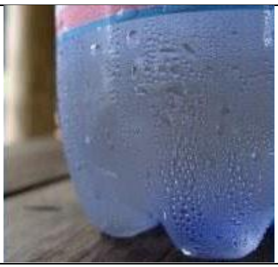	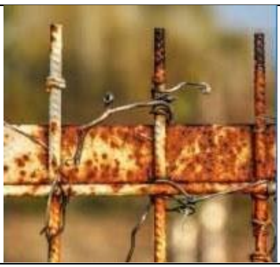	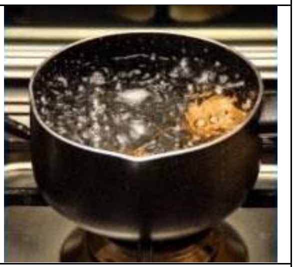
1	2	3	4

84. olfaktorisch			
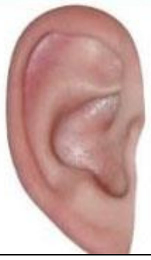	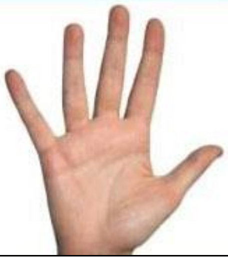	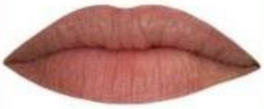	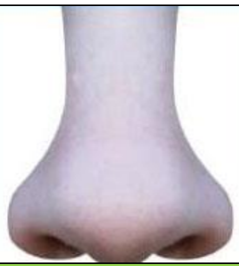
1	2	3	4

85. Rhizom			
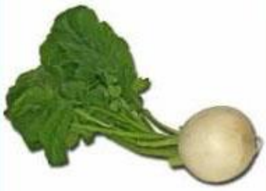	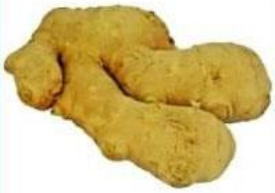	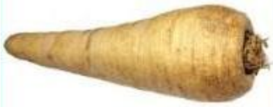	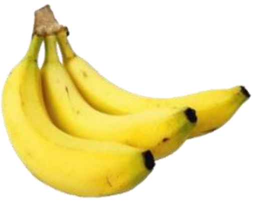
1	2	3	4
