# Supplementary material for: The German Auditory and Image (GAudI) vocabulary test: A new German receptive vocabulary test and its relationships to other tests measuring linguistic experience
Source: PLoS One. 2025 Apr 28;20(4):e0318115. doi: 10.1371/journal.pone.0318115 (PMC12036901; doi:10.1371/journal.pone.0318115)
Supplement: S2 Table — Frequency designations: Zipf_Freq = Zipf frequency obtained from SUBTLEX-DE [49]; HK_Leipzig = Häufigkeitsklassen (frequency classes) obtained from Projekt Deutscher Wortschatz (Project German Vocabulary) of Leipzig University [51]; dwds_freq = frequency level obtained from Digitales Wörterbuch der deutschen Sprache (DWDS) [52]; dwds_hits = number of tokens in the DWDS corpus [52]. (PDF) [file pone.0318115.s002.pdf]

	item	Zipf_freq	HK_Leipzig	dwds_freq	dwds_hits
1	Barett	1,66	21	1	9745
2	Fassade	3,30	11	3	792996
3	Ametropie	NA	NA	0	236
4	Kormoran	1,83	17	2	41479
5	biwakieren	2,13	20	1	2252
6	ekstatisch	2,36	17	2	31699
7	konzentrisch	NA	21	1	16227
8	Triade	2,31	19	1	9394
9	Grummet	NA	24	0	679
10	Wirbel	3,30	12	3	218133
11	Bolide	1,83	15	2	42428
12	Paläontologe	2,59	17	2	19966
13	gelappt	NA	23	1	4978
14	prokrastinieren	NA	20	1	2695
15	Organigramm	NA	17	2	25531
16	kandieren	NA	19	1	6604
17	apportieren	2,40	19	1	7842
18	Emission	1,83	15	3	333307
19	Spachtel	2,20	18	2	33086
20	opulent	1,83	16	2	115864
21	Metronom	2,05	16	2	20605
22	Schwaden	2,43	18	1	13534
23	Pistolengriff	NA	22	1	3157
24	Habit	1,66	19	1	10747
25	Perkussion	1,83	17	2	22863
26	degustieren	NA	18	1	8992
27	urban	2,31	17	3	402139
28	Philatelie	NA	18	1	15373
29	seihen	NA	21	1	10303
30	Galionsfigur	2,13	16	2	25439
31	gastronomisch	1,83	17	3	164380
32	palpieren	NA	NA	0	383
33	Arkade	NA	19	2	52191
34	Artefakt	3,28	16	2	83710
35	posterior	NA	23	1	2070
36	Hellebarde	NA	19	1	7959
37	klandestin	1,66	19	1	6152
38	lanzettförmig	NA	NA	0	1135
39	sinnieren	1,66	17	2	83507
40	Rosette	2,20	18	2	32727
41	verschmähen	2,26	18	2	43385
42	kumulieren	NA	19	2	28636
43	Wehr	3,20	12	3	954603
44	Gefälle	2,50	14	2	90780
45	konferieren	1,66	18	1	13960

46	Aversion	2,13	17	2	19324
47	Büste	2,72	15	2	64811
48	Neonat	NA	NA	0	41
49	Pagode	2,98	18	2	30212
50	Ampere	1,83	15	2	40614
51	Halm	2,68	17	2	51039
52	konisch	NA	20	2	63676
53	Feuchtgebiet	NA	17	2	50008
54	invertebrata	NA	NA	0	21
55	Erker	NA	17	2	35086
56	Konfekt	2,47	19	1	10591
57	laben	2,61	17	2	30208
58	Degression	NA	20	1	5168
59	Safran	3,06	15	2	52350
60	prekär	2,13	14	3	218126
61	Mammalogie	NA	NA	0	174
62	Zenturio	2,68	20	1	1774
63	Kolonie	3,17	13	3	219655
64	Konvoi	3,21	13	2	69804
65	baufällig	2,05	16	2	53206
66	Kazoo	NA	20	1	2563
67	treuhänderisch	1,83	17	2	22818
68	Bimsstein	1,66	20	1	6884
69	Eruption	2,36	14	2	36190
70	Couturier	1,83	19	1	11315
71	indigniert	NA	20	1	2916
72	geothermal	NA	24	1	3617
73	verbarrikadiert	2,68	16	2	25595
74	dressieren	2,43	20	1	12708
75	Diwan	1,96	17	1	10942
76	schelmisch	2,13	17	2	30764
77	Piaffe	NA	19	1	3712
78	pektoral	NA	NA	0	138
79	Bukett	1,66	20	2	21917
80	Irrigation	1,66	21	0	1026
81	Tilde	1,83	20	1	3575
82	Animosität	1,66	20	2	16812
83	Sublimation	1,66	20	1	4321
84	olfaktorisch	NA	20	1	14657
85	Rhizom	NA	20	1	13195
