# Supplementary material for: The German Auditory and Image (GAudI) vocabulary test: A new German receptive vocabulary test and its relationships to other tests measuring linguistic experience
Source: PLoS One. 2025 Apr 28;20(4):e0318115. doi: 10.1371/journal.pone.0318115 (PMC12036901; doi:10.1371/journal.pone.0318115)
Supplement: S3 Table — Frequency designations for cues and targets: Zipf_freq = Zipf frequency obtained from SUBTLEX-DE [49]; HK_Leipzig = Häufigkeitsklassen (frequency classes) obtained from Projekt Deutscher Wortschatz (Project German Vocabulary) of Leipzig University [51]; dwds_freq = frequency level obtained from Digitales Wörterbuch der deutschen Sprache (DWDS) [52]. (PDF) [file pone.0318115.s003.pdf]

	cue	target	Zipf_freq cue	Zipf_freq target	HK_Leipzig cue	HK_Leipzig target	dwds_freq cue	dwds_freq target
practice	Lärm	Stille	3,98	3,80	11	12	3	3
practice	anführen	folgen	3,35	4,80	13	9	3	4
practice	modern	altmodisch	3,56	3,42	13	15	4	2
1	flüstern	schreien	3,61	4,39	17	13	2	3
2	Individuum	Kollektiv	3,14	2,90	14	12	3	3
3	säen	ernten	2,82	3,35	15	13	2	3
4	Beleidigung	Kompliment	3,57	3,77	12	13	3	3
5	atheistisch	religiös	1,66	3,28	20	14	2	3
6	Lüge	Wahrheit	4,44	5,06	12	10	3	4
7	variabel	konstant	1,83	2,99	14	11	3	3
8	lebendig	tot	4,21	5,39	12	10	3	4
9	Zwerg	Riese	3,73	3,22	15	14	3	3
10	öffentlich	privat	3,73	3,77	9	11	4	4
11	global	lokal	3,54	3,55	12	12	4	4
12	billig	teuer	3,85	4,25	12	9	3	4
13	Fusion	Trennung	3,35	3,74	12	10	3	3
14	Freund	Feind	5,39	4,51	9	11	4	3
15	geizig	großzügig	3,20	3,87	17	12	2	3
16	Fiktion	Realität	2,95	4,14	14	10	2	3
17	Verbraucher	Produzent	2,47	3,81	9	12	4	3
18	viel	wenig	5,79	5,10	5	7	5	4
19	Fälschung	Original	3,60	3,96	14	11	3	3
20	eng	weit	4,04	5,19	9	7	4	5
