# Supplementary material for: The German Auditory and Image (GAudI) vocabulary test: A new German receptive vocabulary test and its relationships to other tests measuring linguistic experience
Source: PLoS One. 2025 Apr 28;20(4):e0318115. doi: 10.1371/journal.pone.0318115 (PMC12036901; doi:10.1371/journal.pone.0318115)
Supplement: S4 Table — Frequency designations for cues and targets: Zipf_freq = Zipf frequency obtained from SUBTLEX-DE [49]; HK_Leipzig = Häufigkeitsklassen (frequency classes) obtained from Projekt Deutscher Wortschatz (Project German Vocabulary) of Leipzig University [51]; dwds_freq = frequency level obtained from Digitales Wörterbuch der deutschen Sprache (DWDS) [52]. (PDF) [file pone.0318115.s004.pdf]

	cue	target	Zipf_freq cue	Zipf_freq target	HK_Leipzig cue	HK_Leipzig target	dwds_freq cue	dwds_freq target
practice	fallen	aufstehen	4,76	4,32	8	13	4	3
practice	draußen	drinnen	5,19	4,48	10	12	4	3
practice	Lob	Kritik	2,43	3,58	11	7	3	4
1	eckig	rund	2,13	3,94	17	5	2	5
2	Täter	Opfer	3,97	4,63	8	8	4	4
3	Lösung	Problem	4,33	5,34	8	7	4	4
4	schmutzig	sauber	3,94	4,59	14	11	3	4
5	stark	schwach	4,85	4,29	7	11	5	4
6	Monolog	Dialog	2,99	3,67	15	10	2	3
7	Zukunft	Vergangenheit	4,87	4,54	7	8	4	4
8	faltig	glatt	2,43	3,87	19	12	2	3
9	heiß	kalt	4,70	4,67	10	10	4	4
10	Ebbe	Flut	2,90	3,55	15	11	2	3
11	pro	contra	4,47	2,47	7	17	4	2
12	Feuer	Wasser	4,91	5,11	8	7	4	4
13	klein	groß	4,65	4,89	10	8	5	5
14	digital	analog	3,57	2,20	11	13	4	3
15	schrumpfen	wachsen	2,70	4,10	13	10	3	4
16	lachen	weinen	4,68	4,42	10	13	4	3
17	Frage	Antwort	5,23	4,73	7	9	5	4
18	reich	arm	4,55	4,68	12	13	4	3
19	betrunken	nüchtern	4,40	3,78	12	13	3	3
20	hell	dunkel	3,96	4,29	12	12	3	4
