# Supplementary material for: The German Auditory and Image (GAudI) vocabulary test: A new German receptive vocabulary test and its relationships to other tests measuring linguistic experience
Source: PLoS One. 2025 Apr 28;20(4):e0318115. doi: 10.1371/journal.pone.0318115 (PMC12036901; doi:10.1371/journal.pone.0318115)
Supplement: S5 Table — Critical parts of the sentence are marked in bold print. Asterisks mark grammatically incorrect sentences. (PDF) [file pone.0318115.s005.pdf]

	Category	Item
practice	Prepositions requiring genitive	*Die Übernachtung in dem Hotel kostet **einschließlich den zwei Hunden** nur 80 Euro.
practice	Indirect speech	Herbert erwähnte, es **ginge** ihm nicht besonders gut.
1	Prepositions requiring genitive	***Laut dem Ministeriumsprecher** wird die Maskenpflicht zum nächsten Monat abgeschafft.
2		*Die Durchfahrt wurde **wegen dem massiven Regen** gesperrt.
3		*Das Baby schlief **während dem Essen** ein.
4		*Nach vielem Hin und Her kaufte sie **statt einem Auto** ein Haus.
5		Der Banker investiert das Geld in grüne Aktien **vorbehaltlich ihrer Zusage**.
6		Ein Weg in die Stadt verläuft **links des Hauses**.
7		Die Bahn verspätete sich **aufgrund eines technischen Defekts**.
8		Die angegebene Miete ist **exklusive der Nebenkosten**.
9	Verbs requiring genitive	*Jeden Mittwoch **gedenken sie dem Mann**, der sie rettete.
10		*Die Staatsanwaltschaft **bezichtigte die Bande dem Diebstahl** mehrerer Goldbarren.
11		*Der arrogante Sportler **spottete dem Vergleich** mit seinem Kontrahenten.
12		*Nach dem Schiffsunglück **harrte sie ihrem Schicksal** auf einer einsamen Insel.
13		Das Stipendium **entledigte sie all ihrer finanziellen Sorgen**.
14		Die dritte Nacht in Folge hatten die Kinder **ihn seines Schlafes beraubt**.
15		Nach kurzem Zweifeln **besann sie sich ihres Auftrags** und fuhr weiter.
16		Der pöbelnde Fußballer wurde letztlich **des Platzes verwiesen**.
17	Subordination vs. Coordination	*Katarina war im Sommer kaum ansprechbar, **weil sie lernte** die ganze Zeit für ihre Prüfung.
18		*Vor jedem Konzert bereitete sich der Sänger vor, **indem er durchging** die Texte.
19		*Der Gutschein fürs Eisessen verfiel, **da sie arbeitet** Tag und Nacht.
20		*Die Veranstalter sagten die Tour ab, **zumal die Tickets verkauften sich** eh schlecht.
21		Die Schriftstellerin sprach nicht von ihrem Buch, **sondern sie erzählte** eine Geschichte aus ihrem Leben.
22		Freitags war nicht viel mit ihm anzufangen, **denn er war** meistens müde.
23		Um gegen den Stress anzukommen, machte sie Atemübungen **oder sie ging spazieren**.
24		Karl hatte große Pläne fürs Wochenende, **weil er nicht auf die Kinder aufpassen musste**.
25	Number agreement on verb	*Die Schüssel mit den roten Streifen **kosten** zehn Euro.
26		*Der Freund der Schwestern **haben** einen Kuchen gebacken.
27		*Beides, Tische und Stühle, **stehen** auf vier Beinen.
28		*Eine Menge an Menschen **kommen** jedes Jahr zum Festival.
29		Der Teller mit den bunten Mustern **steht** im Schrank.
30		Die Lehrerin der Kinder **spricht** Englisch und Deutsch.
31		Beides, Mützen und Handschuhe, **schützt** vor Kälte.
32		Die Mehrzahl der Studierenden **kommt** zur Versammlung.
33	je-desto; als-wie	***Desto** besser es der Wirtschaft geht, **desto** höher ist der Lebensstandard.
34		***Umso** länger ich darüber nachdenke, **desto** unsicherer bin ich mir.
35		*Lisa hat auf dem Zeugnis bessere Noten **wie** Paula.
36		*Die Joggerin läuft genauso schnell, **als** die schwedische Leichtathletin bei der letzten Olympiade lief.
37		Je größer die Stadt ist, **desto** höher fällt die Lärmbelästigung aus.
38		Je mehr Philip arbeitet, **umso** höher ist sein Verdienst.
39		Das Auto wurde genau so ausgestattet **wie** die vorherigen Modelle.
40		An der Veranstaltung nahmen mehr Kolleginnen **als** im Vorjahr teil.
41	Indirect speech	*Sabine sagte, sie **kauft** morgen das Geschenk im Einkaufszentrum.
42		*Tom erwähnte, dass seine Freundin sich **verspätet**.
43		*Julius hatte keine Lust mehr auf Schule und wünschte sich, dass schon Ferien **sind**.
44		*Sebastian träumte, er **ist** ein Astronaut.
45		Stefan erzählte, er **habe** morgen eine Verabredung im Kino.
46		Tanja merkte an, ihr Freund **komme** später von der Arbeit zurück.
47		Sybille äußerte gegenüber ihrer Mutter, dass sie gerne eine Ausbildung zur Yogalehrerin machen **würde**.
48		Jonas wünschte sich, er **tränke** ein Bier mit den Rolling Stones.
