# Supplementary material for: The German Auditory and Image (GAudI) vocabulary test: A new German receptive vocabulary test and its relationships to other tests measuring linguistic experience
Source: PLoS One. 2025 Apr 28;20(4):e0318115. doi: 10.1371/journal.pone.0318115 (PMC12036901; doi:10.1371/journal.pone.0318115)
Supplement: S6 Table — Infit and outfit mean square values between 0.5 and 1.5 are considered productive for measurement, while values below 0.5 are deemed less productive, but not degrading. (PDF) [file pone.0318115.s006.pdf]

	item	S_X2	df.S_X2	RMSEA.S_X2	p.S_X2	outfit	z.outfit	infit	z.infit
1	Barett	22,88	16	0,05	0,12	0,90	-0,47	1,00	0,04
2	Fassade	9,77	2	0,15	0,01	0,90	0,00	0,97	0,06
3	Ametropie	21,94	22	0	0,46	0,96	-0,36	0,97	-0,41
4	Kormoran	13,39	11	0,04	0,27	0,84	-0,56	0,98	-0,06
5	biwakieren	36,54	26	0,05	0,08	0,96	-0,58	0,97	-0,51
6	ekstatisch	4,73	8	0	0,79	1,00	0,10	1,03	0,22
7	konzentrisch	10,06	13	0	0,69	0,92	-0,23	1,02	0,20
8	Triade	1,31	5	0	0,93	0,76	-0,52	1,00	0,11
9	Grummet	49,70	26	0,07	0,00	1,29	3,43	1,22	3,34
10	Wirbel	5,21	6	0	0,52	0,92	-0,08	1,00	0,10
11	Bolide	34,00	23	0,05	0,07	1,12	1,22	1,05	0,64
12	Paläontologe	8,60	7	0,04	0,28	1,12	0,44	1,03	0,21
13	gelappt	26,13	26	0,01	0,46	1,08	1,06	1,04	0,73
14	prokrastiniere	NA	NA	NA	NA	0,48	-0,30	0,96	0,27
15	Organigramm	7,80	11	0	0,73	0,89	-0,33	0,96	-0,14
16	kandieren	10,62	10	0,02	0,39	1,01	0,12	1,05	0,31
17	apportieren	13,47	13	0,01	0,41	0,86	-0,56	0,98	-0,04
18	Emission	14,69	13	0,03	0,33	0,89	-0,44	0,98	-0,05
19	Spachtel	NA	NA	NA	NA	0,62	-0,21	1,01	0,24
20	opulent	12,13	16	0	0,74	1,00	0,06	0,99	-0,04
21	Metronom	2,75	4	0	0,60	0,99	0,12	0,99	0,08
22	Schwaden	46,51	26	0,07	0,01	0,90	-1,67	0,92	-1,55
23	Pistolengriff	59,94	26	0,09	0,00	1,22	3,01	1,19	3,33
24	Habit	18,74	25	0	0,81	1,02	0,28	1,01	0,29
25	Perkussion	23,83	19	0,04	0,20	0,85	-0,82	0,95	-0,39
26	degustieren	18,49	24	0	0,78	0,81	-2,18	0,88	-1,86
27	urban	6,01	8	0	0,65	0,80	-0,57	0,97	-0,04
28	Philatelie	15,62	26	0	0,94	0,92	-1,33	0,93	-1,39
29	seihen	27,26	25	0,02	0,34	0,96	-0,49	0,95	-0,78
30	Galionsfigur	28,27	26	0,02	0,35	0,85	-2,17	0,87	-2,40
31	gastronomisch	2,28	3	0	0,52	0,97	0,12	1,02	0,17
32	palpieren	20,39	24	0	0,67	0,94	-0,60	0,96	-0,66
33	Arkade	18,63	18	0,01	0,42	1,17	0,94	1,07	0,55
34	Artefakt	6,48	6	0,02	0,37	1,14	0,47	1,06	0,29
35	posterior	16,96	20	0	0,66	0,84	-1,14	0,90	-1,00
36	Hellebarde	18,45	26	0	0,86	0,93	-1,00	0,96	-0,73
37	klandestin	33,66	26	0,04	0,14	1,09	1,28	1,05	0,99
38	lanzettförmig	11,27	15	0	0,73	0,84	-0,76	0,93	-0,46
39	sinnieren	24,63	22	0,03	0,32	0,86	-1,13	0,97	-0,31
40	Rosette	21,74	22	0	0,48	0,94	-0,47	0,96	-0,39
41	verschmähen	9,90	11	0	0,54	0,86	-0,46	1,02	0,15
42	kumulieren	10,14	13	0	0,68	0,85	-0,71	0,98	-0,08
43	Wehr	29,68	25	0,03	0,24	1,12	1,62	1,12	1,99
44	Gefälle	9,05	6	0,06	0,17	0,98	0,09	1,01	0,12
45	konferieren	9,44	11	0	0,58	0,77	-0,84	0,99	0,01

46	Aversion	17,44	13	0,05	0,18	0,79	-1,00	0,95	-0,28
47	Büste	12,62	6	0,08	0,05	0,56	-1,26	0,95	-0,09
48	Neonat	17,54	26	0	0,89	0,87	-1,89	0,90	-1,80
49	Pagode	34,44	26	0,04	0,12	1,09	1,35	1,08	1,44
50	Ampere	NA	0	NA	NA	0,60	-0,37	1,01	0,21
51	Halm	8,29	8	0,01	0,41	1,12	0,45	1,02	0,16
52	konisch	27,62	20	0,05	0,12	0,76	-1,68	0,89	-1,06
53	Feuchtgebiet	4,14	4	0,01	0,39	1,39	0,89	1,05	0,25
54	invertebrata	24,58	25	0	0,49	0,88	-1,46	0,92	-1,28
55	Erker	13,53	13	0,02	0,41	0,85	-0,57	0,98	-0,06
56	Konfekt	12,49	20	0	0,90	1,00	0,06	0,98	-0,15
57	laben	20,57	26	0	0,76	0,85	-2,40	0,87	-2,59
58	Degression	14,71	10	0,05	0,14	1,36	1,18	1,01	0,12
59	Safran	6,70	8	0	0,57	0,74	-0,81	0,98	-0,03
60	prekär	12,80	13	0	0,46	1,16	0,71	1,05	0,32
61	Mammalogie	24,08	24	0,00	0,46	0,91	-0,87	0,94	-0,78
62	Zenturio	24,64	21	0,03	0,26	0,82	-1,37	0,91	-0,88
63	Kolonie	22,68	20	0,03	0,30	0,96	-0,20	0,95	-0,43
64	Konvoi	11,64	7	0,06	0,11	0,56	-1,32	0,95	-0,12
65	baufällig	3,02	3	0,01	0,39	0,68	-0,52	0,99	0,10
66	Kazoo	14,16	26	0	0,97	1,01	0,23	1,00	-0,02
67	treuhänderiscl	8,21	11	0	0,69	0,83	-0,58	1,01	0,10
68	Bimsstein	25,11	20	0,04	0,20	0,91	-0,54	0,96	-0,33
69	Eruption	12,73	11	0,03	0,31	0,62	-1,52	0,92	-0,35
70	Couturier	30,06	25	0,03	0,22	1,03	0,47	1,03	0,52
71	indigniert	31,42	26	0,04	0,21	1,11	1,73	1,09	1,67
72	geothermal	18,69	13	0,05	0,13	1,00	0,06	1,02	0,16
73	verbarrikadier	5,96	10	0	0,82	0,85	-0,44	0,99	0,00
74	dressieren	38,58	26	0,05	0,05	1,16	2,21	1,13	2,21
75	Diwan	22,08	26	0	0,68	0,79	-3,40	0,82	-3,50
76	schelmisch	6,02	7	0	0,54	0,64	-1,02	0,96	-0,05
77	Piaffe	23,22	23	0,01	0,45	1,03	0,29	1,05	0,62
78	pektoral	24,25	24	0,01	0,45	1,00	0,03	1,02	0,31
79	Bukett	14,33	13	0,02	0,35	0,71	-1,39	0,92	-0,48
80	Irrigation	31,13	26	0,03	0,22	1,09	1,43	1,07	1,29
81	Tilde	19,13	24	0	0,74	0,90	-0,94	0,97	-0,40
82	Animosität	25,79	26	0	0,47	0,99	-0,19	1,00	-0,06
83	Sublimation	35,47	26	0,05	0,10	1,16	2,07	1,08	1,34
84	olfaktorisch	24,66	24	0,01	0,42	0,77	-2,46	0,85	-2,22
85	Rhizom	33,08	26	0,04	0,16	1,00	0,05	1,01	0,14
