# Supplementary figures and images for: The German Auditory and Image (GAudI) vocabulary test: A new German receptive vocabulary test and its relationships to other tests measuring linguistic experience
Source: PLoS One. 2025 Apr 28;20(4):e0318115. doi: 10.1371/journal.pone.0318115 (PMC12036901; doi:10.1371/journal.pone.0318115)

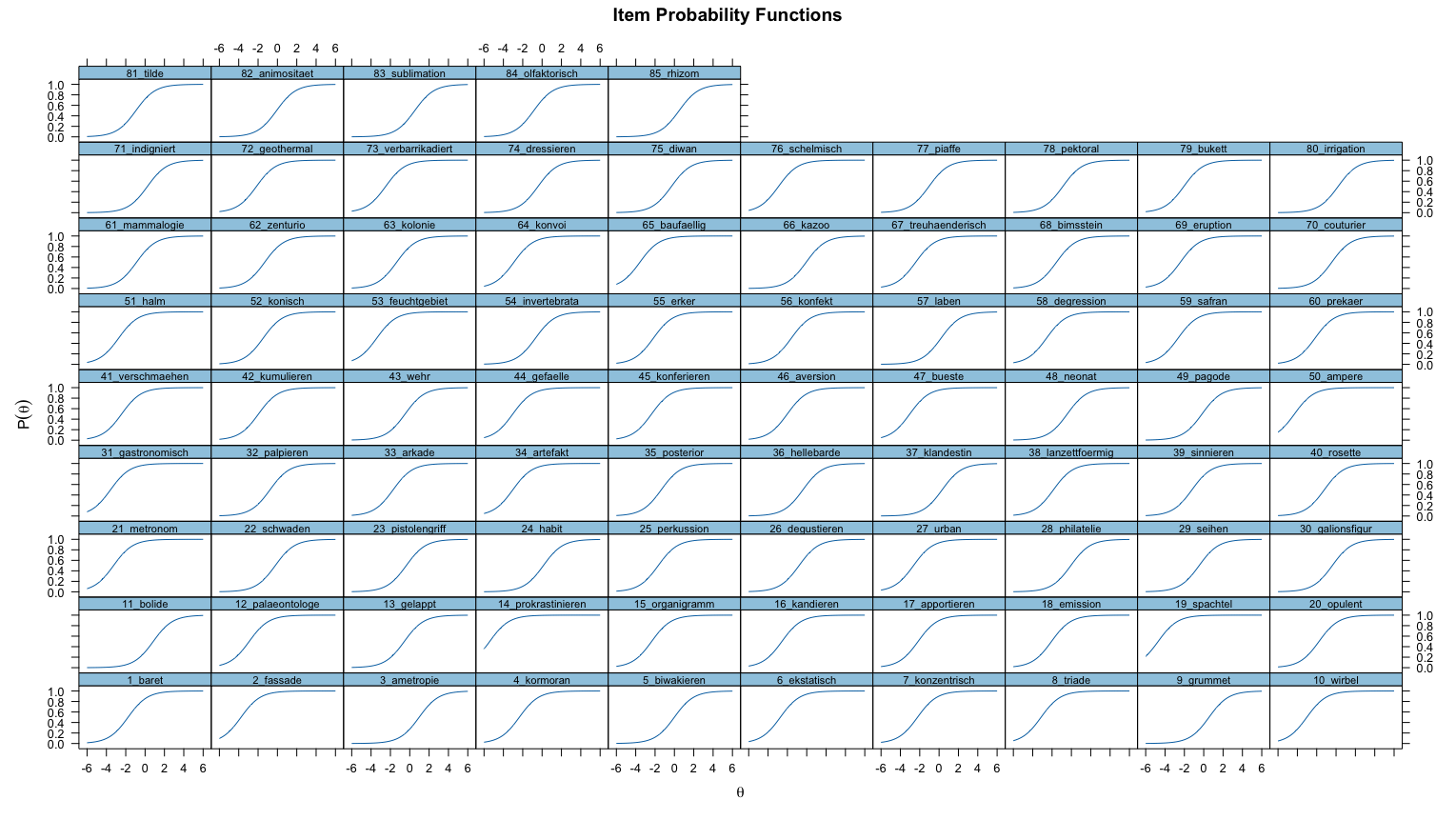

Supplement: S7 Fig — Item characteristics curves for each item in the GAudI. (TIF) [file pone.0318115.s007.tif]
